# Direct Bottom-Up *In Situ* Growth: A Paradigm Shift for Studies in Wet-Chemical Synthesis of Gold Nanoparticles

**DOI:** 10.1021/acs.chemrev.2c00914

**Published:** 2023-06-06

**Authors:** Gail A. Vinnacombe-Willson, Ylli Conti, Andrei Stefancu, Paul S. Weiss, Emiliano Cortés, Leonardo Scarabelli

**Affiliations:** † BioNanoPlasmonics Laboratory, CIC biomaGUNE, Basque Research and Technology Alliance (BRTA), Donostia-San Sebastián, Gipuzkoa 20014, Spain; ‡ NANOPTO Group, Institut de Ciència de Materials de Barcelona, Bellaterra, Barcelona 08193, Spain; § Nano Institute Munich, Faculty of Physics, Ludwig-Maximilians-University Munich, Königinstraße 10, 80539 Munich, Germany; ⊥ Departments of Chemistry and Biochemistry, Bioengineering, and Materials Science and Engineering, University of California, Los Angeles, Los Angeles, California 90095, United States; ¶ California NanoSystems Institute, University of California, Los Angeles, Los Angeles, California 90095, United States

## Abstract

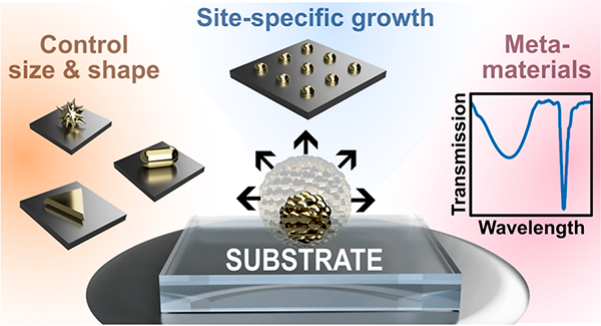

Plasmonic gold nanoparticles have been used increasingly in solid-state systems because of their applicability in fabricating novel sensors, heterogeneous catalysts, metamaterials, and thermoplasmonic substrates. While bottom-up colloidal syntheses take advantage of the chemical environment to control size, shape, composition, surface chemistry, and crystallography of the nanostructures precisely, it can be challenging to assemble nanoparticles rationally from suspension onto solid supports or within devices. In this Review, we discuss a powerful recent synthetic methodology, bottom-up *in situ* substrate growth, which circumvents time-consuming batch presynthesis, ligand exchange, and self-assembly steps by applying wet-chemical synthesis to form morphologically controlled nanostructures on supporting materials. First, we briefly introduce the properties of plasmonic nanostructures. Then we comprehensively summarize recent work that adds to the synthetic understanding of *in situ* geometrical and spatial control (patterning). Next, we briefly discuss applications of plasmonic hybrid materials prepared by *in situ* growth. Overall, despite the vast potential advantages of *in situ* growth, the mechanistic understanding of these methodologies remains far from established, providing opportunities and challenges for future research.

## 1 Introduction

Light–matter interactions in metallic nanostructures have been of interest for many years. The origins of the optical properties of noble metal nanoparticles such as gold, silver, and copper are found in the presence of high densities of free conduction electrons, which approximate a “solid-state plasma”.^1^ When electromagnetic radiation interacts with these systems, collective motion of the free-electron gas results in high electromagnetic field confinement over volumes smaller than the wavelength of the impinging light, accompanied by remarkable near-field enhancements.^2^ This collective motion, known as plasmon resonance, has strong effects on the scattering and absorption behavior of these classes of materials, enabling a wide variety of novel applications in photo- and electrocatalysis,^3,4^ biomedicine,^5,6^ photovoltaics,^7^ imaging,^8−10^ electronic devices,^11,12^ and sensing.^13−16^ The optical properties of coinage metals attract particular attention for their spectral position throughout the visible and infrared (IR) regions of the electromagnetic spectrum.^17−19^ Specifically, the spectral position of the plasmon band of individual plasmonic nanoparticles depends on their size, shape, and local dielectric environment.^20^ The most common strategy for modifying the plasmonic response of individual nanoparticles is to do so in colloidal suspensions *via* bottom-up syntheses.^21−24^ Silver offers the highest performance among noble metals for the generation of strong individual plasmonic resonances in the visible spectral region,^25,26^ while aluminum shows a strong plasmonic response in the ultraviolet (UV), and both elements have been utilized for lasing, optoelectronics, and chemical catalysis.^27,28^ However, gold is the benchmark for bottom-up synthesis and the mechanistic understanding of colloidal growth, owing to its chemical stability.^29^ For these reasons, gold will be the main focus of this Review.

More than simply tuning individual nanoparticles, controlling their positions relative to one another can be used to engineer more complex, cooperative plasmonic responses. When two or more nanoparticles are placed in close proximity, their localized plasmon modes interact, yielding new resonance modes and different distributions of the associated electric field.^30−33^ Moreover, arrangement of particles into chains or periodic arrays with set spacings can lead to hybridization between the individual plasmon modes and the diffraction modes of the array, producing lattice-plasmon resonances.^13,34−36^ Even more interesting magnetic, electronic, and optical responses can be engineered *via* fabrication of different patterns^37−41^ such as gratings,^42^ waveguides,^43−45^ and chiral superstructures.^46−49^ Finally, while some colloidal systems can be engineered to exhibit complex plasmonic responses,^40,50−56^ many devices, sensors, heterogeneous catalysts, and biomedical platforms benefit from incorporating plasmonic nanostructures on or within solid supports such as polymers,^57−59^ glass,^60−63,5,6^ hydrogels,^64,65^ semiconductors,^66−70^ two-dimensional (2D) materials,^71−73^ and metals.^74^

Both top-down and bottom-up nanofabrication approaches have been applied to engineer plasmonic substrates. Although top-down methods (*e.g.*, electron beam evaporation, focused ion beam lithography, electro- and thermal deposition) benefit from their control over the positions of the particles and the generalizability of the substrates,^38,46,75,76^ they suffer from the requirement of clean-room facilities and costly specialized equipment. Techniques based on bottom-up chemical synthesis, on the other hand, such as templated,^13,35−37,40,77^ capillary-assisted,^78,79^ optically printed,^80,81^ and electrophoretic self-assembly,^82,83^ offer economical and accessible alternatives.^75,76,78,84,85^ The desire to apply bottom-up synthesis is motivated by the capability to tune the morphology, crystal structure, and surface chemistry of the particles to produce higher quality plasmon resonances.^86,87^ Despite these advantages, plasmonic substrate fabrication beginning from colloidal building blocks often involves multistep, time-consuming self-assembly, and presynthesis processes that limit their scalability. For instance, bulk colloidal synthesis requires precise temperature and addition rate control over liter-scale reactions.^88,89^ Furthermore, self-assembly often involves modification of the nanoparticle surface chemistry with the addition of large excesses of capping ligands and multiple centrifugation steps.^35,36,48^ The development of a facile, scalable, and rapid technique that bridges the uniformity, precision, and flexibility of top-down methods with the chemical control and affordability of bottom-up methods would greatly improve access to finely tuned plasmonic substrates tailored to desired applications.

**Figure 1:**
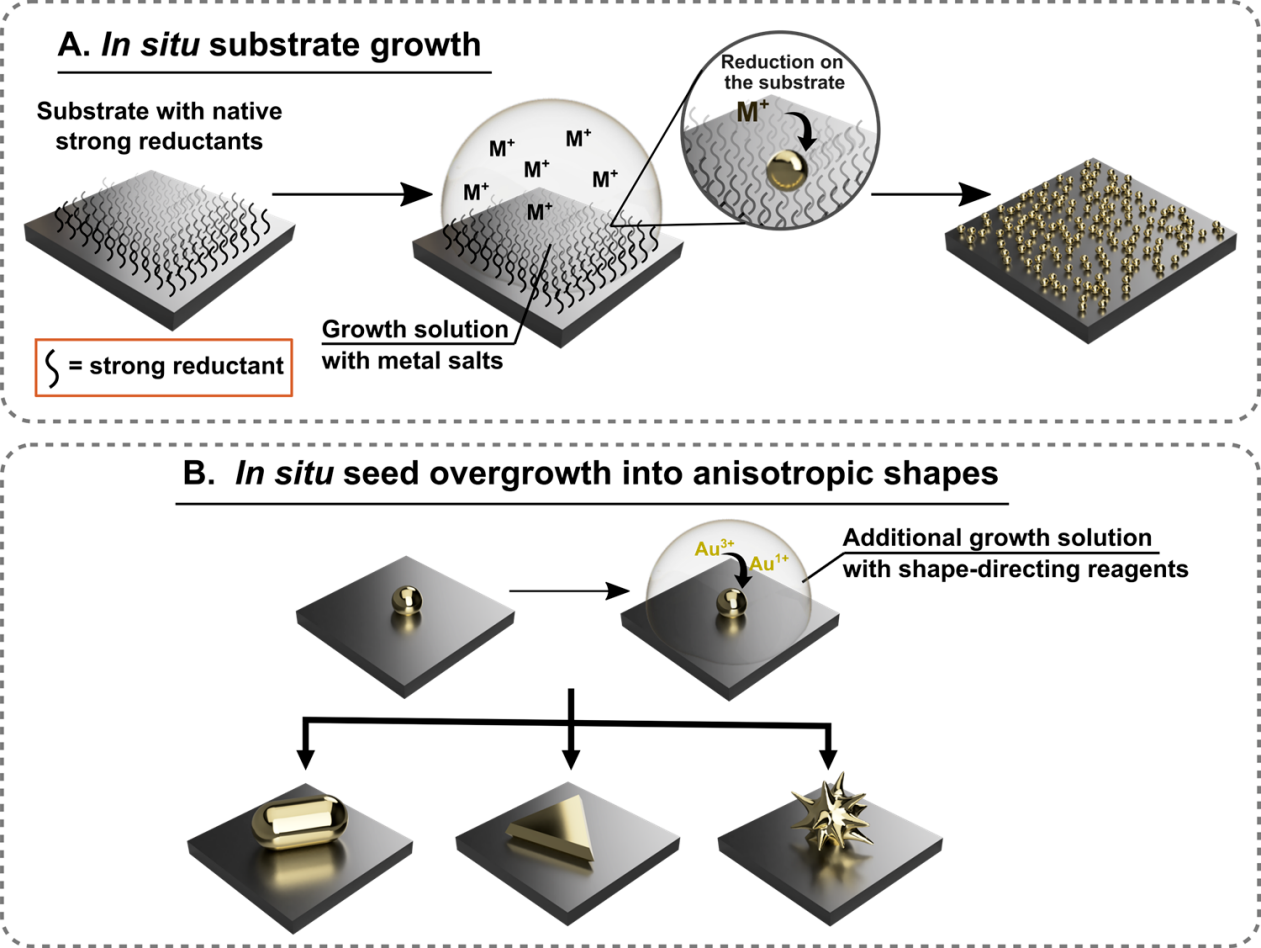
(A) Routes for direct *in situ* chemical surface growth of metal nanoparticle nuclei. (B) Controlled overgrowth of the nuclei, or seeds, on the substrate (*in situ* shape modification).

*In situ growth* is an unconventional bottom-up approach where plasmonic structures, such as size- and shape-controlled nanoparticles, are chemically formed starting from metallic ions directly on the target substrate material without any initial colloidal synthesis steps (Figure 1). Since nanoparticles are formed directly on the substrate, self-assembly, ligand exchange, and other time-consuming processes can potentially be circumvented. In this Review, we focus on the *in situ* growth of scalable and versatile gold metasurfaces, discussing properties, unconventional and *in situ* fabrication techniques, and applications.

The first section of the Review provides a brief general description of the physical and optical properties of plasmonic nanoparticles, nanoparticle assemblies, and plasmonic metasurfaces and metamaterials. This section provides the motivation and background for the main body of the Review, in which we comprehensively discuss the synthetic advances of *in situ* growth methods and the establishment of patterning techniques based on *in situ* strategies, specifically focusing on gold nanomaterials. Because this Review primarily discusses the synthesis and growth of nanostructures on surfaces, following the background on plasmonics, we also provide a brief introduction focused on the history and development of colloidal nanosynthesis (Section 2.1). In Section 3, potential applications of plasmonic substrates, primarily plasmonic metamaterials, are elaborated. Despite recent advances, there are many challenges that must be addressed before bottom-up *in situ* growth of plasmonic metamaterials could represent a solid alternative to top-down and colloidal self-assembly. In the final section, we highlight future research directions beyond the state-of-the-art, anticipating that it will stimulate concerted efforts to enable the accelerated progress using this (to date) unconventional fabrication method.

### 1.1 Plasmonic Properties of Metal Nanostructures

Light–matter interactions are governed by Maxwell’s equations, which describe the relationship of electromagnetic fields with the properties of the material, express through its dielectric permittivity (ε) and magnetic permeability (χ). The dielectric permittivity (or dielectric function) of a metal is a complex function of the frequency, generally expressed as:
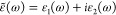
1and is directly related to the refractive index of the medium , also shaped as a complex function of the frequency as: 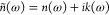
2These two relationships can be combined into: 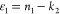
3and
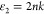
4leading to
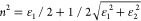
5and 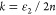
6

The real part (*n*) is related to the dispersion of the electromagnetic radiation in the medium, while the imaginary part (*k*), also known as the extinction coefficient, describes the optical absorption (*i.e.*, the optical losses) of the system. This latter quantity is related to the absorption coefficient α of the Beer–Lambert law
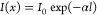
7where *l* is the optical path, describing the exponential decay and attenuation of the intensity (*I*) of a beam propagating in a medium, by the relation^90^
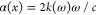
8

An effective representation of the conductive electrons’ behavior is pictured by the Drude model, proposed at the beginning of the 20th century, and based on the application of the kinetic theory to the conduction band of a metal. Briefly, the electrons are treated like a cloud of negatively charged particles, while the positively charged particles (combination of atomic nuclei and valence electrons) are considered fixed. The number of electrons (per unit volume) *N* that can move freely in space is subjected to an external field with harmonic form *E*(*t*) = *E*_0_exp(−*i*ω*t*), and whose oscillations are collision-damped with frequency γ = 1/τ. The external field drives the in-phase oscillation of the electrons, generating the macroscopic polarization *P̅* = −*Nex̂*, where *x̂* is the direction of oscillation, linked to the electric field displacement (*D*) as *D̅* = ε_0_*E̅* + *P̅*. It results in: 
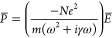
9and
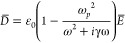
10where 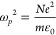
11is the plasma frequency for *N* electrons with effective mass *m*.

Finally, the dielectric function in the free electron gas model can be written as:
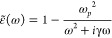
12Consequently, the real and imaginary parts of the dielectric function can be derived from combining eqs 1 and 12 to obtain:
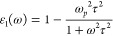
13
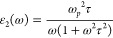
14One can identify two regimes, depending on the frequency of the electromagnetic radiation interacting with the system. At higher frequencies (ω > *ω*_*p*_), ε̃(ω) is mainly real and the phase mismatch generates a retardation that allows the driving electric field component to penetrate the surface of the metal.^91^ This condition concretely means that the conductive electrons of the metal do not interact with the incoming electromagnetic radiation. Instead, when ω < *ω*_*p*_, the real part ε_1_(ω) is negative, and the coherent electron oscillation arrives in phase with the incident radiation. The dielectric function is dominated by ε_2_ > ε_1_ and the metal is mostly absorbing.

In this latter case, following interactions with impinging electromagnetic radiation, the metallic object can reradiate the incident field by scattering the incoming light, and convert part of its energy to different forms following absorption.^92^ The system is said to extinguish the incident light, as a result of both scattering and absorption processes whose relation can be expressed by the cross-section quantity: *C*_ex_ = *C*_a_ + *C*_s_. When nanoparticles are characterized by dimensions smaller than (typically below 1/10th of) the electromagnetic radiation wavelength, the elastic scattering can be described using Rayleigh theory, following a wavelength dependence of 1/λ^4^. When the dimensions of the nanoparticles become comparable to the wavelength of the light, instead, the scattering properties are better described by the Mie theory, used to calculate the amount of scattered light by a particle at given directions, breaking them into spherical harmonics, with strong dependence on size and shape. Depending on the geometry of the metal object, we can identify different kinds of plasmon resonances, which are described below.

### 1.2 Surface Plasmon Polaritons

**Figure 2:**
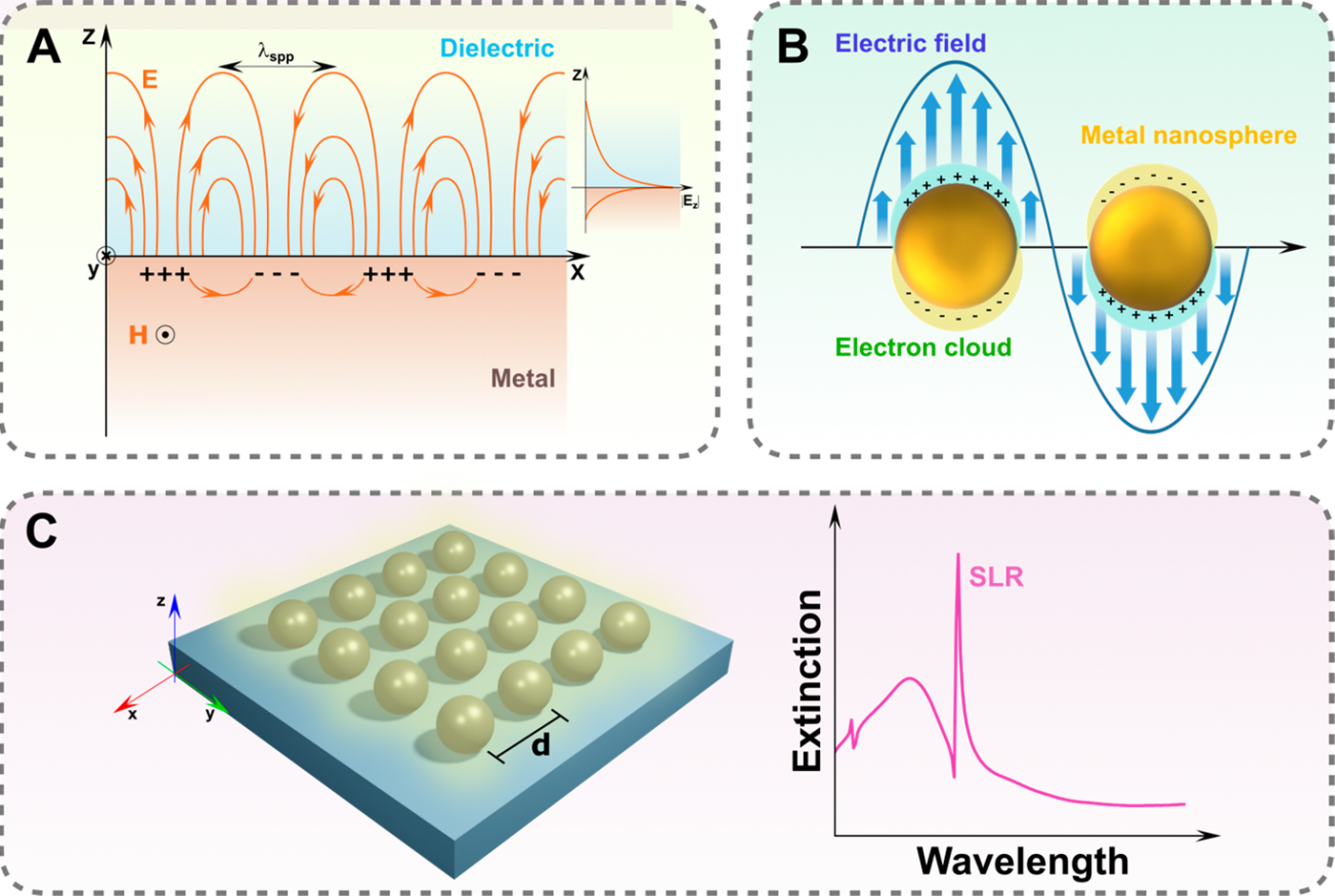
(A–C) Schematics of a (A) surface plasmon polariton at a metal-dielectric interface, (B) localized surface plasmon of a metal nanoparticle, and (C) plasmonic surface lattice resonance (SLR) of a plasmonic array with periodicity *d*. The extinction spectrum on the right exemplifies the narrow bandwidth typically associated with surface lattice resonances.

Surface plasmon polaritons (SPPs) are an extensively studied class of plasmon resonances generated when a metallic surface is in contact with a dielectric medium such as air or glass. They propagate at the metal/dielectric interface decaying evanescently in both media along the direction of propagation (Figure 2A).^93^ The supported electromagnetic wave is bound to the interface (as described from the dispersion relation), where the mobile charges available on the metal surface can be excited in collective motion at frequency 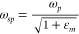
15where *ω*_*p*_ is the plasma frequency and *ε*_*m*_ is the dielectric constant of the environment.

### 1.3 Localized Surface Plasmon Resonance

When electromagnetic radiation of amplitude *|E*_*0*_*|* interacts with a nanosized metal object, such as a nanoparticle, a dipole moment is induced, proportional to the object polarizability α as *p̅ = αE̅*. Considering that the particles are smaller than the incident wavelength, these systems can be treated with a quasi-static approach where the particles’ parameters are frequency dependent and 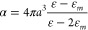
16where *ε*_*m*_ is the environment dielectric constant (Figure 2B). Physically, the induced coherent motion of the free conduction electrons results in a resonance highly localized around the nanometric volume, due to the increase of the polarizability α (when Re [ε(ω)] = −1/2*ε*_*m*_ for the Fröhlich condition).^94^ These types of oscillations are known as localized surface plasmon resonances (LSPR) and, as mentioned above, for noble metals, fall into the visible-near-IR range of the electromagnetic spectrum. One of the great advantages of these plasmonic modes arises from their exceptional intensity and spectral tunability as a function of the particle size, shape, and composition.^95^ Moreover, when two or more nanoparticles are placed in close proximity, the interactions of their plasmon modes can be described using a model similar to molecular orbital hybridization. A nanoparticle’s individual plasmon modes hybridize with one another to produce low-energy bonding and higher-energy antibonding plasmon modes.^30−33^ The interactions depend on the reciprocal orientation of their associated dipoles, leading to coupling of their localized plasmon bands and resulting in significant changes in the optical properties of the system.^2^ In these small gaps, the spatial distribution of the “bonding” plasmon modes similarly resembles bonding molecular orbitals and high electric field enhancements, and localization in the near-field region (below 20 nm) can be recorded, leading to the formation of the so-called “hot spot” regions.^96−100^ These highly localized electromagnetic fields have been exploited for the enhancement of weak optical signals, with the development of several ultrasensitive detection techniques such as surface-enhanced Raman spectroscopy (SERS), surface-enhanced infrared absorption (SEIRA), and plasmon-enhanced fluorescence (refer to Section 3.3 for an expanded discussion of these applications).

### 1.4 Plasmonic Surface Lattice Resonances

By arranging nanoparticles into periodic arrays, with periods comparable to the wavelength of the incident electromagnetic radiation, a new type of plasmon resonance emerges when the in-plane scattered field of each dipole unit arrives in phase with the scattered field of the neighboring particles in the array. The LSPR of each coupled nanoparticle in the array hybridizes with incident light diffractive orders at wavelengths close to the Rayleigh-Wood anomalies, giving rise to collective optical responses, called surface lattice resonances (SLRs),^101−105^ as shown in Figure 2C.

The collective response driven by the incident electromagnetic field is due to the electrical dipole induced in the particles, which is able to radiate along the orthogonal direction, where it can couple to the wave vectors of the diffraction orders. When a plane wave with wave vector *k*_0_ impinges on an array surface, the light is scattered in the plane by all particles, with wave vector *k*. As presented by Guo et al., the scattering vector *s̅* is the result of the difference between *k* and *k*_0_, and the amplitude of the scattered wave is proportional to ∑*l* exp (*is̅* · *r̅*_*l*_), where for a 2D array, *r̅*_*l*_ = *n*_1_*a̅*_1_ + *n*_2_*a̅*_2_ is the direct lattice vector with its primitive vectors *a̅*_1_ and *a̅*_2_.^106^ The conditions for which the total scattered amplitude is nonzero result when *s* equals the reciprocal lattice vector G̅ = *m*_1_*b̅*_1_ + *m*_2_*b̅*_2_, where *b̅*_1_ and *b̅*_2_ are primitive vectors of the reciprocal lattice (diffraction orders). If more particles are considered in a unit cell, an exponential term, the so-called envelope factor *r*_b_, which modifies the magnitude and phase of the scattering signal, should be added.^106^

In the diffraction condition, the dispersion relation of the Bragg diffraction orders, which describes these lattice modes on the base of the empty lattice approximation, can be written as:
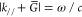
17with *n*_eff_ as the effective refractive index and *k*_//_ as the wavevector component parallel to the lattice surface. For the case of a 2D square array, if in each direction of the plane (*xy*) the lattice vectors have the same magnitude, at *k*_//_ = 0 (*i.e.*, at normal illumination incidence), the diffractive orders are degenerate, addressable to the same Γ point of the Brillouin zone. Specifically, the spectral position (*λ*_RW_) of the Rayleigh-Wood anomalies can be predicted using the following simplified equation:
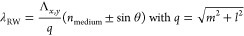
18where *n*_medium_ is the refractive index of the grating surroundings. In the majority of cases, only the first diffraction order is considered (⟨ ± 1,0⟩ and ⟨0, ± 1⟩, implying *q* = 1). Eq 18 shows how SLRs are spectrally tunable by varying the size of the plasmonic units and/or the array lattice period.^107^

The tunability of the narrowband resonances across the electromagnetic spectrum can be exploited by employing elastomers as substrates. For instance, Fery and co-workers have demonstrated that particles assembled into ordered arrays on a PDMS substrate are able to provide a reversible transformation of the array under strain, which shifts the resonance over 70 nm.^108^ Moreover, SLRs can provide strong near-field enhancement, high scattering cross sections, spatial delocalization, and longer lifetimes, depending on the physicochemical properties of the composing plasmonic units and on the polarization of the incident light.

The resulting SLRs are delocalized over the entire periodic array. Since the array extends in both *x* and *y* directions, the number of dipoles added to the far-field interaction scales with the second power of the distance, effectively compensating for the decay and consequently reducing the optical losses of the resonance compared to the 1/*d*^3^ decay length characteristic of near-field interactions. In this way, it is possible to increase the lifetime of SLRs, leading to the appearance of narrow spectral features (Figure 2C). The figure of merit used to quantify the effect of the optical losses is known as quality factor *Q*_f_ = λ/Δλ, where λ is the resonance wavelength and Δλ is its width. Compared to LSPRs, which typically exhibit *Q*_f_ values below 10, SLRs are characterized by high *Q*_f_ (>100), which increases proportionally to the number of NPs composing the array.^109^ Engineering SLRs for different applications is discussed in Section 3.1. In the following section, bottom-up synthetic approaches for fabricating plasmonic gold nanomaterials are discussed.

## 2 From Colloidal Synthesis to *In Situ* Growth

### 2.1 Colloidal Synthesis of Gold Nanoparticles

The most common strategy for tuning the plasmonic properties of nanoparticles is to do so in colloidal suspensions *via* bottom-up synthesis. We focus in this Review on gold plasmonic nanoparticles in particular, consistent with the majority of the literature, and so this section includes colloidal synthetic developments in this area. There are three basic elements in the colloidal wet-chemical synthesis of gold nanoparticles: (*i*) the gold salt, the source of gold ions, usually HAuCl_4_, NaHAuCl_4_, or KAuCl_4_, (*ii*) a capping ligand, usually a surfactant, to stabilize the particles in colloidal suspension *via* Coulombic and/or steric repulsion, and (*iii*) a reducing agent to reduce the Au^3+^ ions. Although there exist approaches where electrochemical reduction^110,111^ and/or light^112,113^ are used in place of a chemical reductant, we will not focus on these approaches here and instead direct readers to recent comprehensive reviews.^114−117^

#### 2.1.1 Seed-Mediated Growth

Although nanoobjects of various dimensions and geometries can be synthesized with one-pot methods,^118−122^ seed-mediated growth is probably one of the most prevalent and well-developed approaches for tuning the size and shape of gold nanoparticles with high yield. The idea for gold seed-mediated growth dates back as early as 1925 and proceeds by heterogeneous nucleation using small “seed” nanoparticles as templates for later overgrowth.^123^ This approach leads to improvements in the size distributions of the products due to control of the chemical environment separately and precisely during the nucleation and growth stages. In this scheme, the chemical environment of the seed solution is optimized so that nuclei are rapidly formed (quasi-simultaneously) *via* secondary nucleation. The uniform seeds are then introduced into a second “growth solution” containing a different composition of gold salt, capping ligand, and reducing agents. Natan and co-workers performed initial explorations into seed-mediated growth for gold nanospheres.^124^ Here, seeds were prepared by rapid reduction of Au^3+^ to Au^0^ by sodium borohydride and boiling aqueous citrate. On the other hand, the growth solution was a mild reducing environment, promoting final reduction from Au^1+^ to Au^0^ to occur directly on the preformed seeds. The implementation of only weak reductants (rather than strong reductants capable of completely reducing Au^3+^ to Au^0^) in the growth solution is key for effectively disfavoring additional secondary nucleation during the growth step, significantly improving uniformity. Interestingly, oblate spheroid products were also produced for certain compositions of the growth solution.^124^ The possibility to select for such anisotropic objects through seed-mediated growth was subsequently explored in great detail. The size and shape of the seed,^125−127^ reductant,^128,129^ pH,^130,131^ temperature,^97,132^ and the presence of “shape-directing reagents”^133−139^ serve to direct the anisotropic growth of gold nanoparticles.^23,24,134−136,138−142^

**Figure 3:**
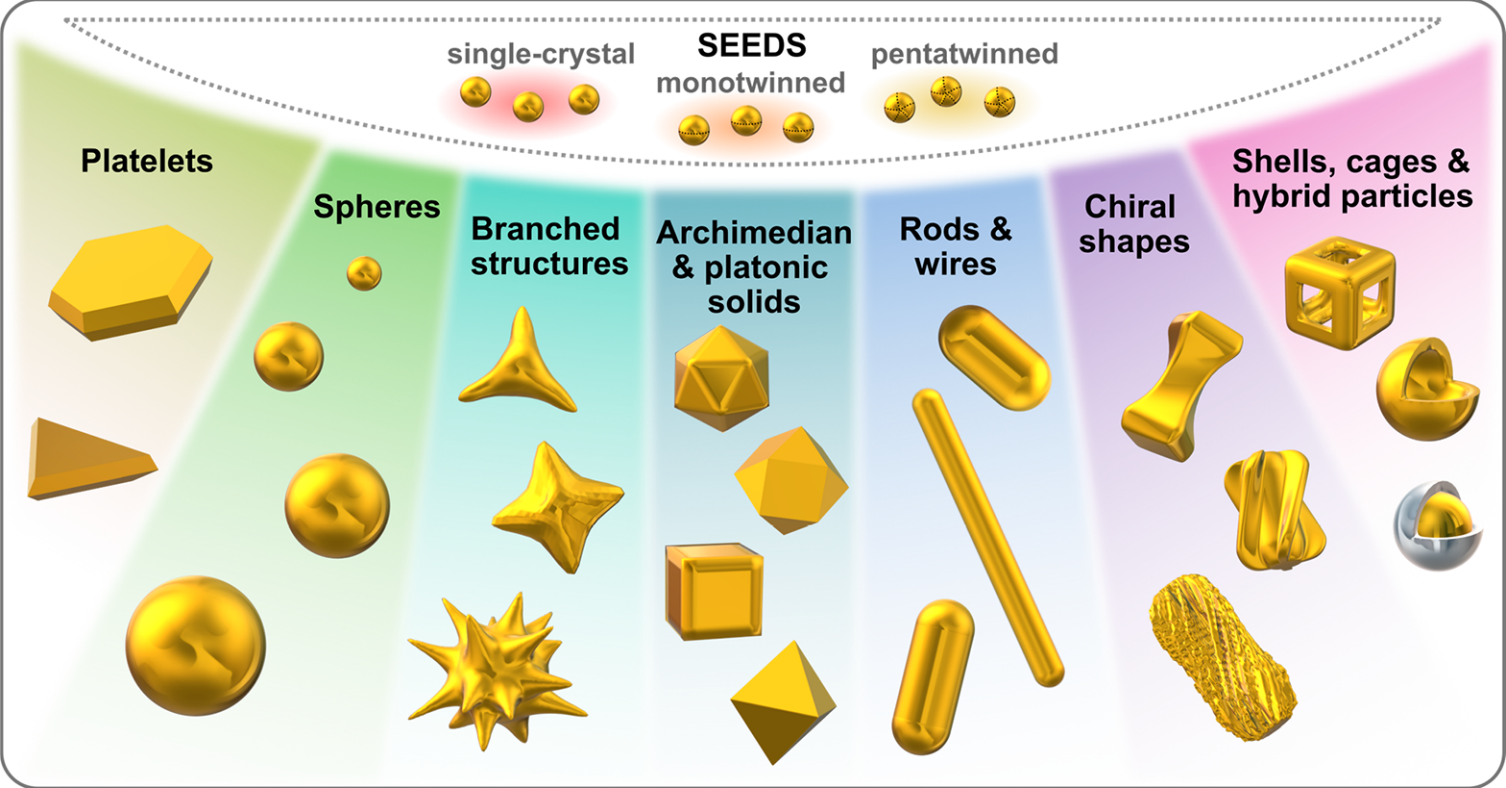
Schematic showing examples of gold nanoparticle seeds and some geometries accessible with bottom-up seed-mediated growth.

Thus far, syntheses of rods^143^ and stars^128,144^ from single crystalline seeds, platelets and triangles from monotwinned seeds,^126,145^ and nanorods,^129^ bipyramids, dodecahedra,^132,146^ penta-branched stars^128,147^ from penta-twinned seeds have been reported. Particles presenting polycrystalline features^148^ and more complex facets (*e.g*., chiral shapes)^149−153^ have also been demonstrated with the seeded growth method. The implementation of multiple growth steps has also been used to make hollow/cage^154,155^ and hybrid nanostructures.^156−158^ In Figure 3, we provide a summary of some of the possible geometries that can be accessed. In the growth solution, as with seed synthesis, the selected reductant, pH, surfactant, halides, solvent, and silver salts all act to promote growth along specific crystal facets.^24^ Undoubtedly, there are many “handles” for controlling shape-directed growth; comprehensive reviews of each of these aspects are available elsewhere.^21−24,140,159^

In addition, colloidal satellite structures or systems studying the growth of nanoparticles on another host nanomaterial, such as nanoparticle-decorated nanoplatelets, nanotubes, or other colloidal supports, offer interesting possibilities for generating hybrid structures, which we discuss further in Section 2.2.^52,160−163^ Furthermore, the translation of the synthetic rules from this literature, especially regarding the promotion of nucleation on the colloidal support, could be informative for further developing synthetic protocols whereby gold nanoparticles are grown on substrates. This strategy can be especially interesting for polymers, where prior work has shown that the synthetic conditions can be varied to alter the degree of embedding of the satellite particles on the supporting colloid.^164−166^

#### 2.1.2 Plasmonic Substrates from Colloidal Nanoparticles

In contrast to top-down methods, bottom-up colloidal syntheses make it possible to fabricate higher quality single-crystal nanoparticles and to tune morphology rationally. Integrating colloidal particles with substrates typically begins with nanoparticle presynthesis, followed by ligand exchange/modification of the particle surface chemistry and self-assembly.^35,97,167−170^ Uniform films of presynthesized gold nanoparticles can be prepared following ligand exchange and self-assembly *via* centrifugation,^171^ controlled solvent evaporation,^15,172−174^ and assembly at liquid–liquid^48,175−177^ and liquid–air interfaces.^126,172,178−180^ These self-assembly processes can be combined with chemically^66,181,182^ or physically patterned^35−37,49,97,167^ substrates, or soft-lithography with nanoparticle inks.^48,64,67,183^ The application of physically patterned stamps and substrates to assist in directing the site-specific attachment of particles is especially popular and includes capillarity-assisted particle assembly^49^ or template-assisted self-assembly.^35−37,49,97,167^ Although there are some reports patterning premade anisotropic particles, such as nanorods^37,184^ and triangles,^185^ it remains challenging to orient and to arrange anisotropic particles on substrates. While soft lithography methods (*e.g.*, microcontact printing,^186−189^ chemical lift-off,^48,190−192^ nanoimprint lithography,^181,193,194^*etc.*) are potentially scalable, the presynthesis, ligand exchange, and self-assembly of the colloids are less so. The latter steps are usually limited by mixing/uneven distribution of reagents in large volumes and the requirement of multiple centrifugation steps. These limitations inspire alternative approaches, where instead of using premade colloids, the metal precursors are reduced directly on the surface and bottom-up chemistry is used to control their shape *in situ*, which is the focus of this Review.

### 2.2 Wet-Chemical *In Situ* Growth

We define wet-chemical *in situ* growth as the bottom-up formation of nanoparticles directly on a solid substrate *via* chemical reduction of gold salts (Figure 1). This process is also sometimes referred to as electroless nanomaterial fabrication or electroless deposition, and for interested readers, more literature regarding *in situ* nanoparticles fabrication can be found using those terms.^195^ However, here we use the terminology “*in situ* surface growth” rather than “electroless deposition” because the specific interest of *in situ* growth is to create nanoparticles with controlled morphology and crystallography *on* the substrate, focusing on interrogating the growth mechanism akin to colloidal size and shape control, but in a new environment. In contrast, electroless deposition is more general and includes metal thin film fabrication.

There are numerous potential benefits to *in situ* synthesis, including: (*i*) the capability to modify size and shape directly on the substrate;^196−200^ (*ii*) chemical control over the particle positions and their orientations relative to the substrate;^201−204^ (*iii*) tunability of the particle–substrate interactions;^205,206^ and (*iv*) the avoidance of time-consuming and complex presynthesis, ligand exchange, and self-assembly. The formation of nanoparticles directly on material surfaces by chemical approaches is not a new idea or practice. In fact, *in situ* formation of gold and other plasmonic metal nanoparticles in colored ceramic glass was routinely performed as early as the 17th century, with early examples, such as the famous Lycurgus cup,^207^ dating back to the fourth century, before there was any idea of the existence of nanoscale particles.^208^ During the glass making process, gold salts and reductant additives such as tin (in the form of stannous chloride) or antimony would be added, leading to the formation of gold nanoparticles within the glass substrate.^207−209^ The particle sizes could be tuned *in situ* by modifying the amount of salt, treatment temperature, and time until the desired intensity and hue were obtained.^209^ Currently, *in situ* growth is being tested and developed for different kinds of materials, either by the addition of strong reductants in nanoparticle growth solutions or by taking advantage of strongly reducing functional groups on the substrate. The potential to use colloidal synthetic principles to modify the particle geometry has also been validated by demonstrations of shape-controlled overgrowth.^196−200^

#### 2.2.1 Promoting Substrate Growth with Chemical Reductants

**Figure 4:**
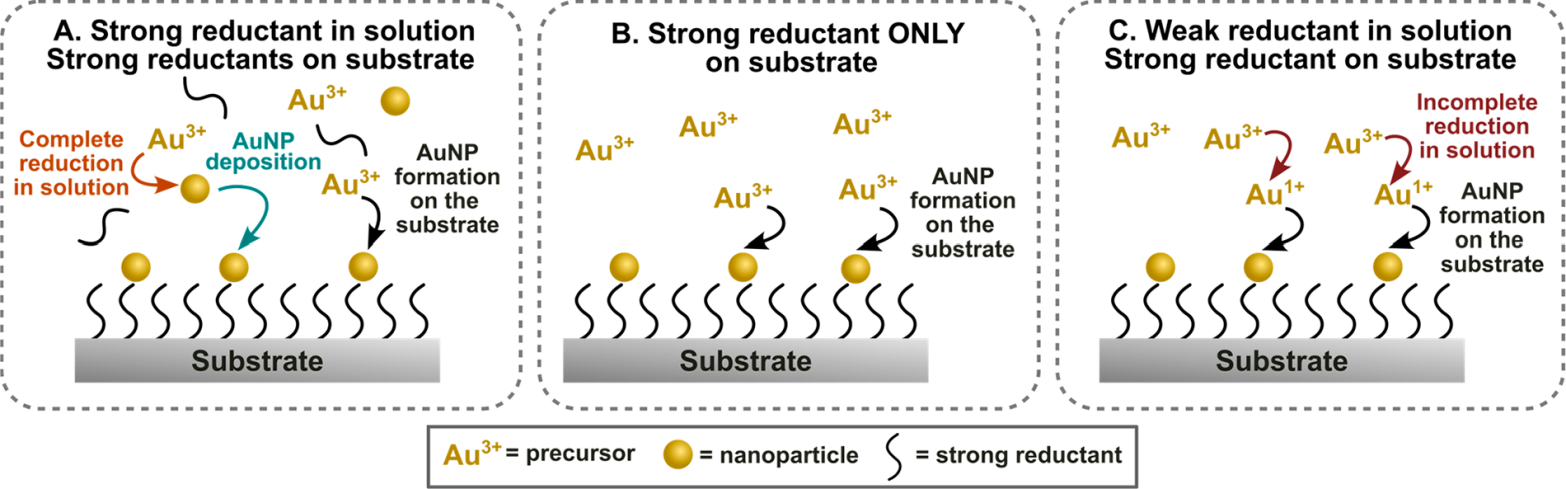
Schematics depicting *in situ* growth with (A) an externally added strong reductant, (B) on a substrate with functionality capable of completely reducing Au^3+^ to Au^0^, and (C) on a substrate with strong reductants, but with weak reducing agents added to the growth solution. AuNP = gold nanoparticle.

As with colloidal synthesis, pure *in situ* growth begins with the initial single-step secondary nucleation of gold nanoparticles from gold salts. In both approaches, the type, strength, and quantity of chemical reductants greatly influences the uniformity and morphology of the final products. Moreover, the timing and conditions under which reductants are introduced (slowly or rapidly, at which temperature, under vigorous stirring, *etc*.) is also important.^88,129,210−212^ First, we focus on the use of chemical reductants, since the method of their application is key for promoting nanoparticle nucleation on the substrate. There are many reports that apply synthetic schemes similar to single-step colloidal synthesis, where a strong reductant is added to a solution containing metal salt and shape-directing reagents (Figure 4A). This strategy has been used to form nanostructures rapidly on substrates such as 2D materials,^213,214^ biocompatible or conductive polymers,^215^ and hydrogels.^216^ However, we note that the presence of strong reductants promotes the formation of colloidal nanoparticles in the growth solution, which competes with the nucleation of nanoparticles on the substrate (Figure 4A). In order to avoid nanoparticle formation and growth in solution, one can instead confine the presence of strong reductants only to the substrate, either by taking advantage of reducing capabilities native to the material or by adding strongly reducing chemical functionality (Figure 4B,C). By taking this route, one can achieve better control over the synthesis, ensuring that there are no competing reactions that contribute to the consumption of growth reagents.

##### 2.2.1.1 *In Situ* Growth on Substrates with Native Ability to Reduce Metal Salts Completely

Strong reductants are not always needed in the growth solution to achieve nanoparticle formation on the substrate. In fact, in many cases, the implementation of a substrate with strong reductants presents advantages compared to adding external strong reductants, because it is less likely that particles will form in solution away from the target substrate (Figure 4B,C). Solution nucleation of particles leads to consumption of the gold precursor and can produce polycrystalline structures that can later deposit onto the surface, increasing polydispersity of the final products.^6,202,217,218^ There are many materials on which direct growth of gold and other metal nanoparticles can be directly performed. A few examples include graphene/graphene oxide,^72,219^ MoS_2_,^71,220^ cellulose,^221,222^ silicon,^223^ polydimethylsiloxane (PDMS),^224−228^ polyelectrolyte multilayers,^229^ and conductive polymers polypyrrole^230^ and polyaniline.^231^

In particular, many studies providing important synthetic insights take advantage of Si–H groups in organosilica, hydridosilica, and other silicone-based polymers.^232−234^ Among these materials, PDMS is highly popular due to its low cost, tunability of its physical properties, and suitability for biological applications.^235−237^ A standard commercial kit for PDMS contains two components, the base and curing agent, which are thoroughly mixed together. Then, thermal treatments are applied to obtain the final polymer. During the heating process, oligosilanes in the base react with the cross-linker polymethylhydrosiloxane from the curing agent solution in the presence of a platinum catalyst. The strongly reducing Si–H groups in native PDMS result from incomplete consumption of polymethylhydrosiloxane.^226,232,234,235^ Some reports explore the synthesis of gold nanoparticles on PDMS, providing important insights into how growth time, temperature, reagent concentrations, and nucleation site density effect nanoparticle morphology.

**Figure 5:**
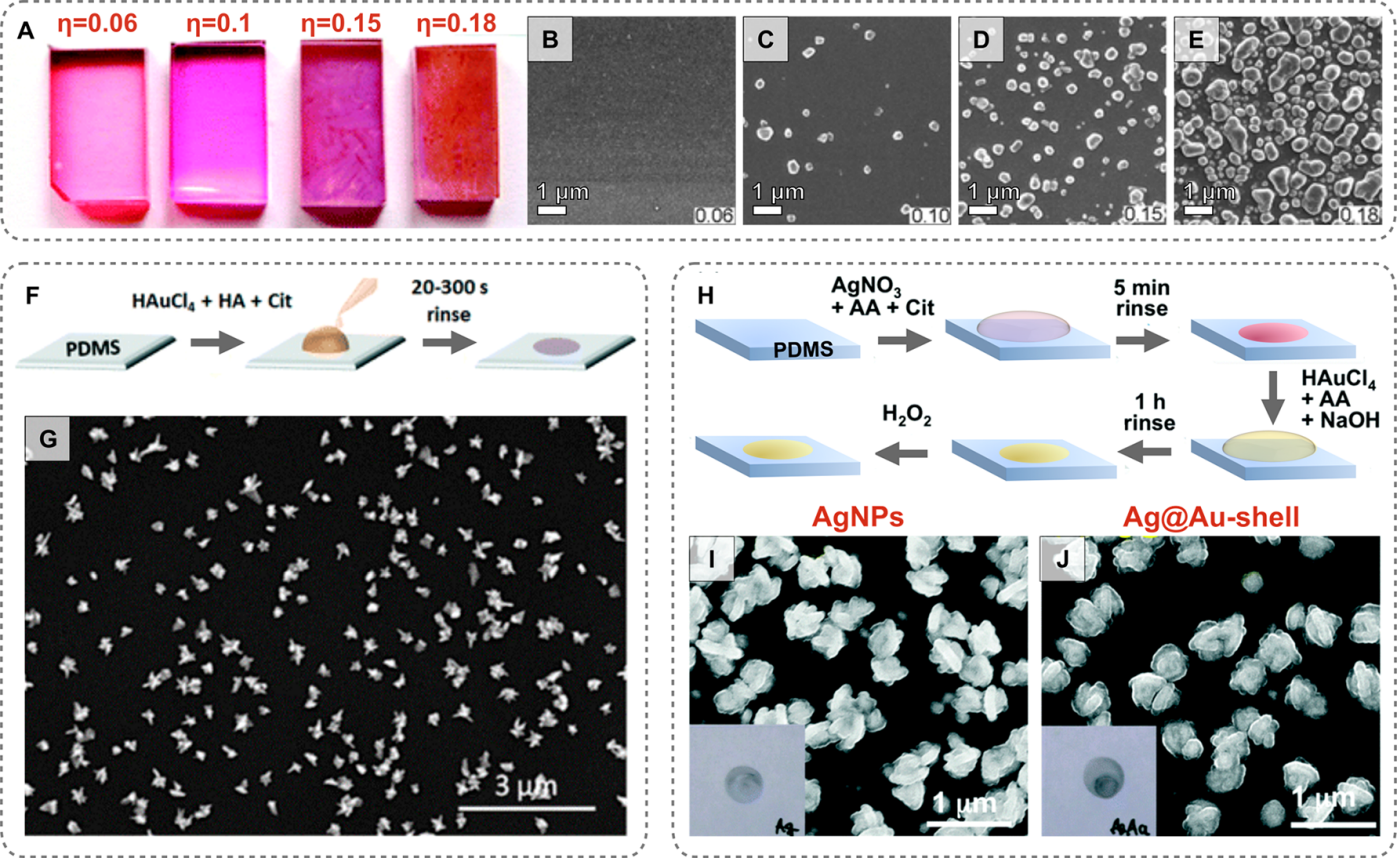
(A) Photographs of polydimethylsiloxane (PDMS) substrates with different curing agent to base mass ratios (η) following gold nanoparticle growth. (B–E) Scanning electron microscopy images of the products [adapted with permission from ref (225). Copyright 2008, Wiley-VCH]. (F) Schematic of the growth process for *in situ* synthesis of citrate-capped nanostars and (G) an electron microscopy image of the products [adapted with permission from ref (59). Copyright 2017 Royal Society of Chemistry]. (H) Schematic of growth process for Ag@Au shell particles and (I,J) images of the synthesized particles [adapted with permission from ref (224). Copyright 2017 Royal Society of Chemistry].

Changing the curing agent-to-base ratio (η) of the PDMS modulates the polymethylhydrosiloxane concentration, therefore modifying the density of Si–H and the nucleation sites. This control can be envisioned as the colloidal synthesis analogue to changing the seed-to-gold-salt ratio. Zhang and co-workers observed that altering the amount of unreacted Si–H could determine whether particle growth was selected to occur within the polymer matrix or localized on the surface.^225^ Moreover, the density of Si–H groups was used to modify the shape and geometry of the prepared nanostructures (Figure 5A–E). A similar result was observed in the work of Bonyár et al., who tested the effects of growth time and temperature by performing the growth at 22–80 °C with incubation times of 2 h to 7 days.^238^ Higher growth temperatures and longer incubation times yielded products with higher extinction, indicative of more growth. One limitation of relying on the native Si–H groups is that their concentration varies with curing time, temperature, humidity, and age of the sample.^235^ However, there are also limits to tuning the η value, because curing agent-to-base ratios that are too low (η < ∼0.5:10) or too high (η ≥ 5:10) produce PDMS films that are no longer structurally robust.^236^ There is also an upper limit to the quantity of nanoparticles that can be incorporated into silicone-based systems before their presence starts to damage or to change the physical properties. In one example, Tsuge et al. observed that too high concentrations of *in situ* silver nanoparticles led to significant increases in the sliding angle of PDMS-based slippery-liquid coatings incorporating polymethylhydrosiloxane as a lubricant.^232^ The effect of nanoparticle density on the material properties is especially important to consider for responsive polymer systems, *e.g.*, poly(*N*-isopropylacrylamide) (PNIPAm).^216^ Moreover, modifying the growth conditions for AuNPs on PDMS was shown to lead to different degrees of particle embedding within the polymer, which was an effect also observed in satellite growth in other polymer systems.^164−166^

In colloidal seed-mediated growth, weak and strong reductants are applied at separate stages of the synthesis, with the seed solution containing the strong reductants and the growth solution containing weak reductants.^24,124^ Strong and weak reductants could also be *spatially* separated for *in situ* growth, with the strong reductants confined to the substrate and weak reductants applied in the growth solution, as depicted in Figure 5F and H. Applying weak reductants along with other shape-directing reagents to the growth solution *in situ* would improve the size and shape yield and facilitate the synthesis of anisotropic shapes. Fortuni et al. tested the separation of strong and weak reductants and the addition of other shape-directing reagents *in situ*, demonstrating substrate growth of gold nanostars and Ag@Au-shells (Figure 5F–J).^59,224^ The *in situ* synthesis of gold nanostars on 1:10 PDMS, with reducing agents hydroxylammonium chloride and citrate, was explored. Within 60 s, particles with diameters of 200 nm were formed and the final stars were obtained after 2 min at a size of ∼300–400 nm. Hydroxylammonium chloride was essential for the rapid growth of nanostars. Furthermore, when neither citrate or hydroxylammonium chloride were added, no nanostars were produced within the 2 min time frame. Compared to relying on the native Si–H groups alone, much lower concentrations of gold salt were used and with shorter synthesis times (2 min *vs* 2 h to several days; 5 mM *vs* 14.7 mM HAuCl_4_). Overall, Fortuni et al. showed that selective formation of nuclei on the substrate is promoted by better incorporating strong reductants on the surface of the target material. Similar to colloidal systems, temperature, reagent concentration, and the presence of shape-directing agents can be used to chemically direct the growth.

##### 2.2.1.2 *In Situ* Reduction of Gold Nanoparticles on Two-Dimensional Materials

Two-dimensional materials are attractive substrates for *in situ* growth functionalization, including transition metal dichalcogenides (TMDs),^239,240^ graphene^73,241,242^ and graphene oxide,^243^ and transition metal carbides (MXenes). These materials have gained increasing interest as electrocatalysts,^244^ photodetectors,^245^ devices for energy conversion and storage,^246^ superconductors,^242^ and magnetic materials.^247,248^ Two-dimensional materials decorated with metal nanoparticles are emerging as novel hybrid systems, where the incorporated nanostructures can be utilized to tune catalytic properties,^249,250^ to act as dopants,^251^ or to identify defect sites.^251^

**Figure 6:**
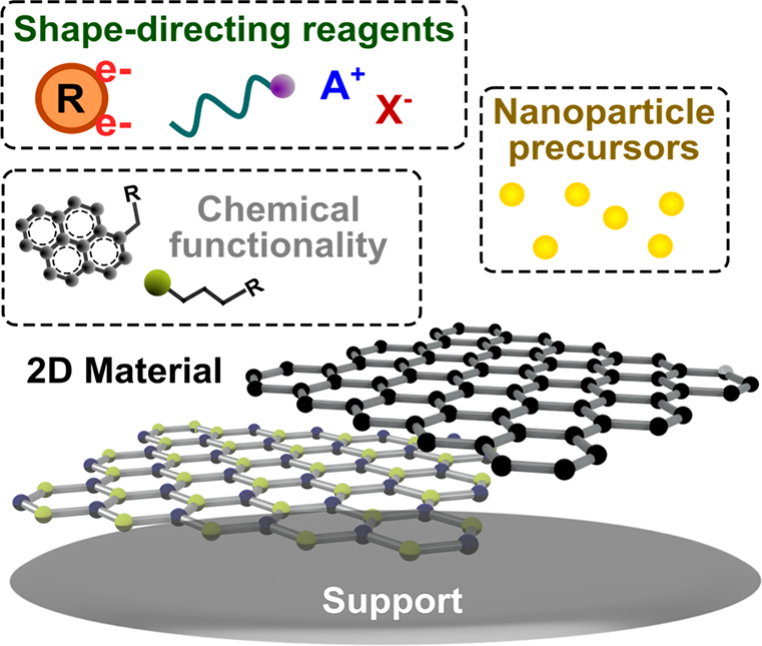
Schematic highlighting the tunable components of *in situ* growth on 2D materials.

For *in situ* growth on 2D materials, different components of the system can be tuned: the nanoparticle type/composition, added reductants, shape-directing reagents, added functionalities on the surface of the 2D-material, and the underlying substrate support (Figure 6). Generally, *in situ* reduction of gold precursors on 2D material proceeds *via* spontaneous electron transfer between the 2D material and metal ions. While this process can occur at pristine regions under the appropriately selected synthetic conditions, metal ion reduction is generally more favorable at defect sites. This reactivity contrast is because only weak van der Waals forces promote interactions with the metal NPs at defect-free areas. Thus, the strategy of creating defect regions in a controlled way (defect engineering) generally results in more uniform and dense metal NP coatings. Aside from gold, platinum,^252^ silver,^253^ alloy particles,^254^ tin oxide, titanium dioxide,^255^ magnetic, and semiconductor^249^ nanoparticles have been formed *in situ* on 2D materials. Reducing agents such as NaBH_4_,^213,214^ sodium citrate,^256,257^ ascorbic acid, glucose (“green” synthesis),^72,258^ ethylene glycol,^259^ 4-nitrophenol,^254^ and hydrazine^253,260^ are commonly used to assist in promoting precursor reduction in these systems. The addition of stabilizers including sodium dodecyl sulfate, sodium citrate, cetyltrimethylammonium bromide (CTAB), polyelectrolytes,^261^ block copolymers,^262^ and polyols have been explored. As with substrates discussed above, the addition of different surface functionality on the 2D material can modify the interaction/density of nanoparticles grown *in situ.* For instance, molecules such as pyrene ethylene glycol amine and decylpyrene can be used to modify graphene-based materials^219,255,260^ and thiols and selenols can be used to add chemical functionalities to TMDs.^249^ Thus, *in situ* growth offers rapid and easy integration of a variety of nanoparticle and 2D material compositions. Even more, studies exploring *in situ* growth on 2D materials can provide fundamental insights on the different mechanisms that promote precursor reduction and nanoparticle formation.

**Figure 7:**
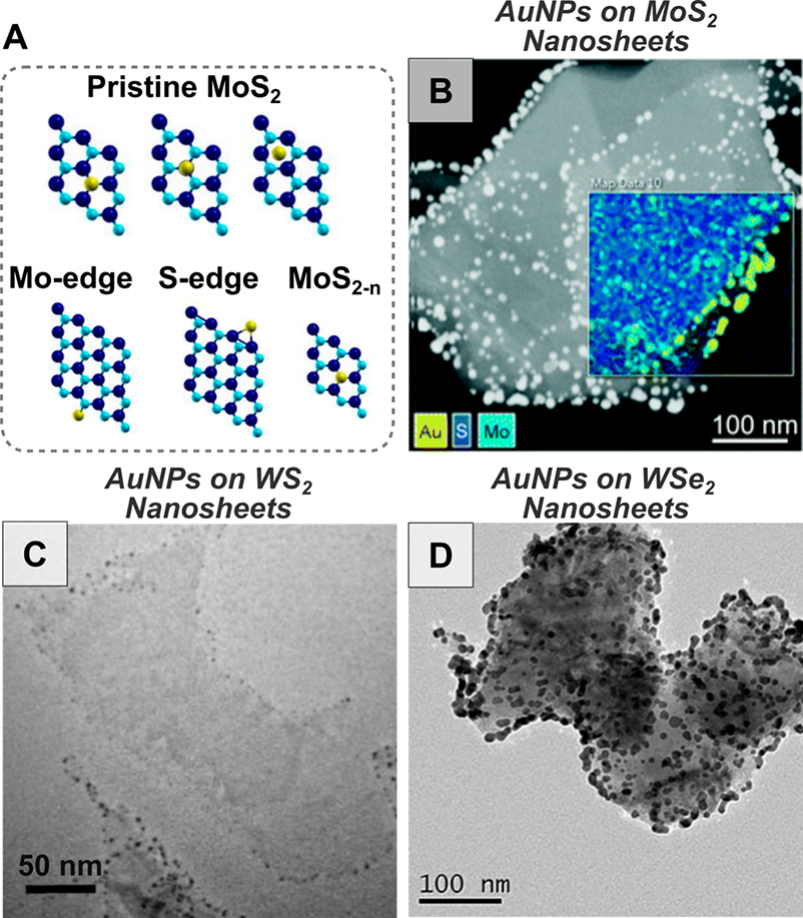
(A) Models showing gold adhesion to different areas on pristine MoS_2_ and at different defect sites [adapted with permission from ref (71). Copyright 2018, Royal Society of Chemistry] (B-D) Electron microscopy of gold nanoparticles (AuNPs) grown on transition metal dichalcogenides: (B) MoS_2_ nanosheets (with elemental mapping), [adapted with permission from ref (71). Copyright 2018, Royal Society of Chemistry] (C) WS_2_ nanosheets [adapted with permission from ref (264). Copyright 2018, Nature Publication Group CC BY-NC-ND 4.0], and (D) WSe_2_ nanosheets [adapted with permission from ref (265). Copyright 2022, Springer Nature].

2.2.1.2.1. *Semiconductor Transition Metal Dichalcogenides*. Semiconductor TMDs are 2D layered materials with composition MX_2_, with M representing a transition metal cation and X a chalcogen atom, with common examples being MoS_2_, WS_2_, and WSe_2_.^263^ In addition to the pristine basal plane of the TMD, defect sites such as thiol/selenol groups and dangling bonds at the edge sites and vacancies promote nucleation (Figure 7A). Most *in situ* growth strategies that incubate WS_2_, MoS_2_, and WSe_2_ nanosheets or nanoribbons with gold or metal salts produce edge- and defect-decorated hybrid materials (Figure 7B–D).

**Figure 8:**
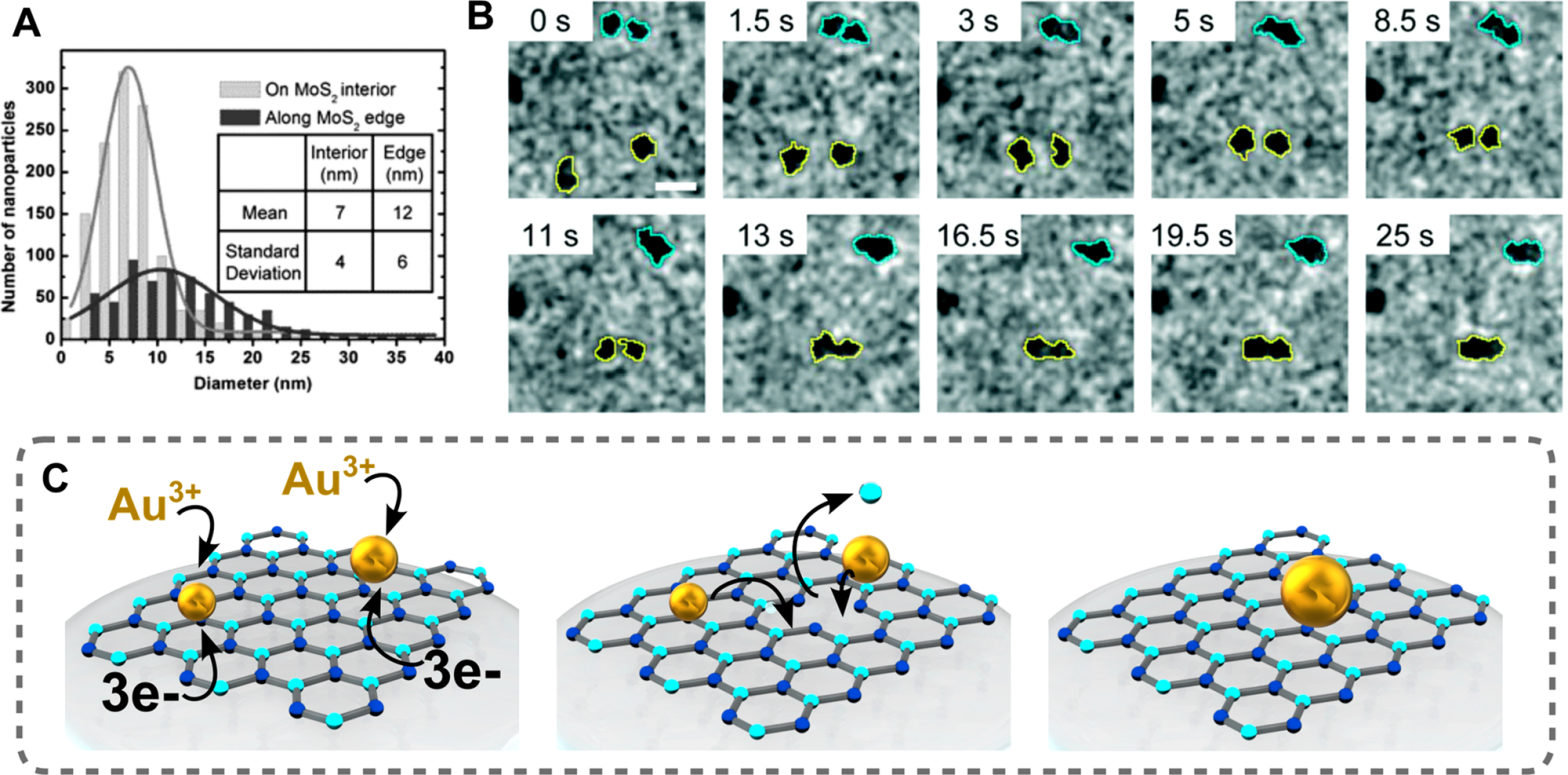
(A) Size distribution of observed nanoparticles at the MoS_2_ interior and at the MoS_2_ edge. (B) Snapshots at different time points of gold nanoparticle growth on MoS_2_. The yellow outline indicates two nuclei that migrate and coalesce [adapted with permission from ref (71). Copyright 2018, Royal Society of Chemistry]. (C) Schematic showing the (I) nucleation, (II) migration to defect sites, and (III) coalescence process.

Song et al. investigated the formation of AuNPs *in situ* with scanning transmission electron microscopy (STEM) to glean more information regarding the nucleation and coalescence of AuNPs on MoS_2_.^71^ While this work mainly focused on *in situ* AuNP growth on MoS_2_ nanoflakes, it provides useful insights into the roles of defect engineering in promoting nucleation and growth on TMDs. The formation of AuNPs was characterized by placing MoS_2_ nanoflakes in a liquid cell consisting of two silicon chips and silicon nitride viewing windows, and subsequently flowing HAuCl_4_ precursor into the chamber to initiate growth. Within 30 s, irregular-shaped particles ranging in size from ∼1 to ∼10 nm were produced, and larger particles were found at the MoS_2_ edge sites compared to the MoS_2_ interior (Figure 8A). Furthermore, Ostwald ripening was observed directly, where the smaller AuNPs dissolved soon after nucleation and the 10s of nm particles instead continued to increase in size, demonstrating that certain phenomena that play roles in colloidal size-focusing are also present in *in situ* systems. Additionally, when sulfur vacancies were added, weakly bound particles on the MoS_2_ interior migrated to those sites and combined with one another during growth (Figure 8B,C).

The preferential nucleation at edge sites and the migration and coalescence of nanoparticles on the basal plane to sulfur vacancies was further supported by density functional theory (DFT) calculations using the Vienna *ab initio* Simulation Package (VASP),^266^ employing the Perdew-Burke-Ernzernhof (PBE) generalized gradient approximation (GGA)^267^ for the exchange-correlation function and projector-augmented wave (PAW) pseudopotentials.^268,269^ The van der Waals interactions between gold and the defect-free MoS_2_ basal plane had an adhesion energy of only 0.66 eV, much lower than at the Mo-edge sites (3.72 eV), S-edge (1.87 eV), and sulfur vacancies (2.77 eV). These results show that tuning the nanoparticle-surface interactions can promote or inhibit AuNP diffusion and coalescence, which is a unique feature of *in situ* systems. In addition, high-angle annular dark-field STEM (HAADF-STEM) analysis showed that Au{111} was preferentially oriented on the basal plane, which the authors attributed to lower lattice mismatches. In contrast, nanoparticles on the edge of the sheets appear to have access to multiple different orientations. This difference is useful to note since understanding how to achieve a specific and uniform crystal orientation is important if later synthetic steps are performed, or if certain crystallographic facets need to be selected for optimal catalytic performance. This work indicates that defect engineering, for instance, the use of hydrogen plasma to generate sulfur vacancies, is important for controlling AuNP nucleation and growth on TMDs. While studies have thus far focused on the effects of nanoparticle nucleation on TMDs, additional studies systematically interrogating the effect of TMD stability and oxidation on AuNP nucleation could provide useful information, considering that TMDs are prone to oxidation (specifically in the 1T metallic phase).

**Figure 9:**
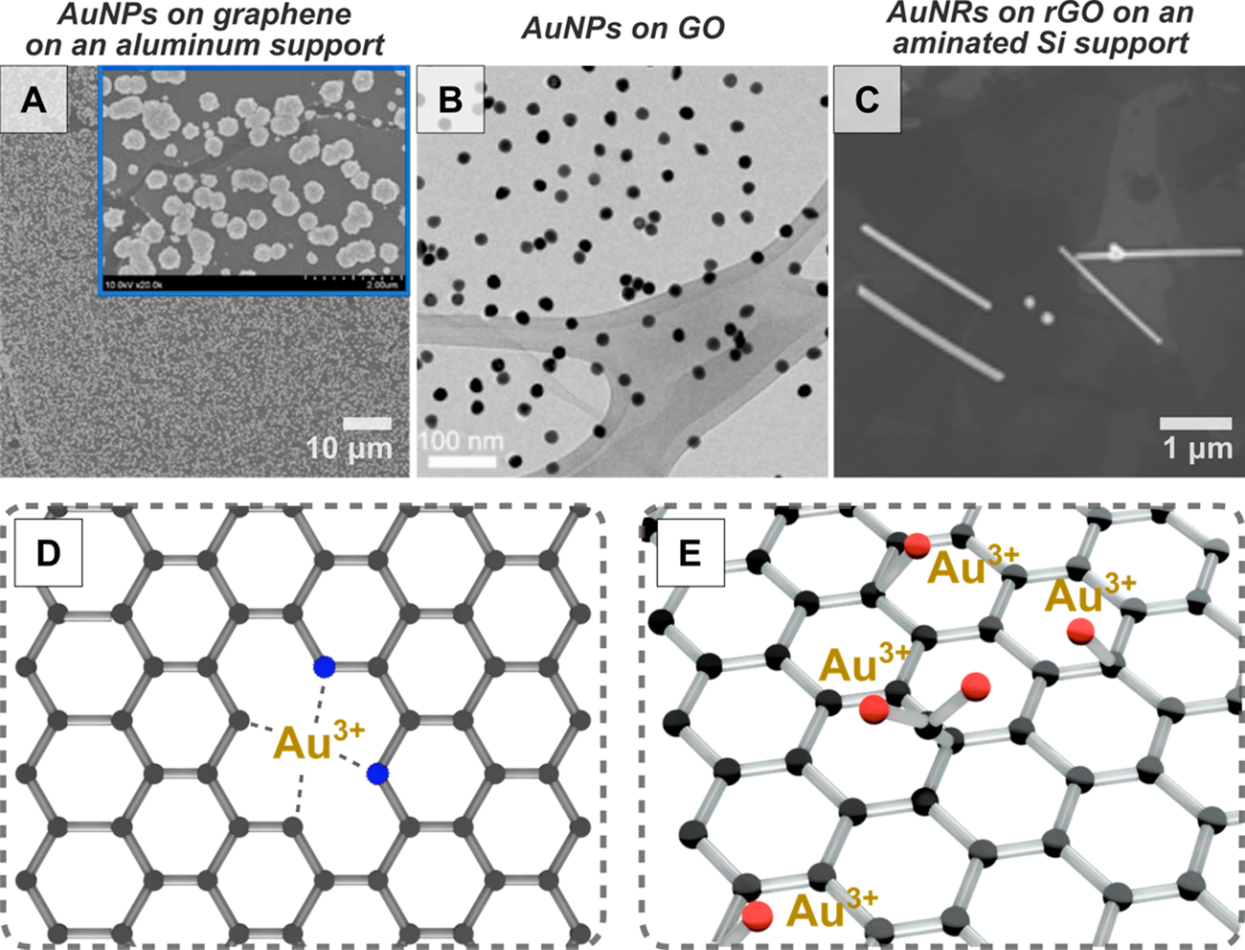
(A–C) Electron microscopy images of nanostructures grown on graphene-based materials. (A) Gold nanoparticles (AuNPs) on graphene on an aluminum support [adapted with permission from ref (271). Copyright 2020 American Chemical Society], (B) AuNPs on graphene oxide (GO) [adapted from ref (276) with permission. Copyright 2013 American Chemical Society], and (C) gold nanorods (AuNRs) on reduced graphene oxide (rGO) supported by an aminated Si substrate [adapted from ref (277) with permission. Copyright 2010 Royal Society of Chemistry.]. (D) Model showing a gold atom interaction with a C_2_N_2_ divacancy pore in nitrogen-treated rGO. (E) Model of oxygen moieties that interact with gold ions in rGO.

2.2.1.2.2. *Graphene-Based Nanomaterials*. As with TMDs, nucleation can also be promoted to occur on pristine graphene,^270,271^ but defect sites are most effective at promoting metal nanoparticle nucleation. Among the different possible defects that may exist in graphene (single-carbon vacancies, multiple-grouped vacancies, impurities, foreign atoms substitution, *etc*.),^214,243,272,273^ carbon vacancies—even at the single atom level—have been proposed as nucleation centers for metal clusters.^274^ Indeed, the binding energy of single to few Pt atoms turns positive when defects are included in the atomistic calculations compared to the pristine graphene case. As such, nucleation centers for further growth can be engineered into graphene by inducing C vacancies in the material. Beyond fully reduced graphene, graphene oxide, reduced graphene oxide,^213,257^ (obtained from the oxidation of graphite and subsequently modified, *i.e.*, through latter thermal and chemical reduction steps) and nitrogen-doped graphene^214,252,262^ (which can be fabricated *via* chemical/hydrothermal reduction of graphene oxide with hydrazine/ammonia)^275^ have been used to create plasmonic nanoparticle-2D material hybrid structures (Figure 9).

For instance, Koo and co-workers explored the formation of AuNPs on rGO with nitrogen-induced defects *via* incubation with gold salt and NaBH_4_ as a reductant.^214^ Multiple growth steps (from 1 to 4) were applied. In the first step, subnanometer particles were synthesized uniformly over the material and after four steps, large, agglomerated nanoparticles were formed. They applied Raman spectroscopy and observed softening of the G-band at 1572 cm^–1^ (in-plane vibrations of *sp*^*2*^ bonded carbon atoms) following *in situ* growth, which is associated with the presence of doping with an electron donor, presumed to be the AuNPs. Density functional theory calculations were performed to estimate the binding energies of gold to divacancy pores using VASP, employing PAW potentials with a plane-wave basis set and the PBE exchange–correlation functional.^266−269^ The calculated binding energies supported that C_4,_ C_2_N_2_, and N_4_ divacancy pores can act as preferential nucleation sites leading to the formation of the subnnanometer gold nanoclusters that were observed (with binding energies of 3.04, 3.14, and 4.99 eV, respectively), which are closer to the cohesive energy of *fcc* gold (3.10 eV) than N_2_O_2_, O_4_, and O_2_C_2_ pores (with binding energies of 0.4 eV. 0.34 eV, and 1.06 eV, respectively). The formation of large agglomerates was reported to occur primarily at defect-free sites on the basal plane due to the weak van der Waals interactions (0.19 eV) between the substrate and gold. Another factor that may have contributed to the appearance of agglomerates is the deposition of particles that formed in solution after the initial nucleation sites were saturated with small nanoclusters, especially considering that the strong reductant NaBH_4_ was used. While they calculated that gold atom binding to oxygen-containing divacancy pores is not thermodynamically favorable, other oxygen-containing moieties present in reduced graphene oxide/graphene oxide (hydroxyl, carboxylate, epoxides) could promote metal nanoparticle nucleation due to electrostatic interactions between the metal ions and the oxygen-containing groups.^213,253,255−257,276^

Similar to Song and co-workers, Goncalves et al. observed that AuNPs grown on substrates appeared smaller and denser with engineered nucleation sites. In this study, more uniform nanoparticles and fewer aggregates were grown on graphene oxide (having more nucleation-promoting oxygen-containing moieties) compared to thermally reduced graphene oxide. Goncalves did not observe any nanoparticle formation on completely reduced graphene, confirming the roles of the oxygen-containing groups in promoting nucleation.^257^ However, it is possible to form AuNPs directly on completely reduced graphene *via* spontaneous galvanic reaction when an underlying supporting material of the graphene is a less noble metal than the one comprising the forming nanoparticles (*i.e.*, aluminum, germanium, or copper, for AuNP growth).^270,271^ In one example, Chang et al. used this reaction scheme and showed that graphene-coated aluminum yielded higher density and uniformity of nanoparticles by the galvanic reaction mechanism compared to *in situ* growth on unprotected aluminum. The increase in quality of the AuNPs was associated with the observation of less secondary nucleation, which accompanied the formation of large aggregates.^271^

After nanospheres, the most common particle shape grown on 2D materials is gold nanorods. The Min group tested various conditions for the synthesis of gold nanorods on graphene. They achieved 37% yield of 2.2–3.5 μm long nanorods with aspect ratios of 28 ± 7 *via* direct synthesis in the presence of shape-directing reagents CTAB and ascorbic acid (AA), a significant improvement over the previously attained 5% yield.^72,219^ Moreover, they showed that functionalization of reduced graphene oxide with pyrene ethylene glycol amine (presenting amines) produced denser products with fewer rods, whereas decylpyrene (with hydrocarbon moieties), produced more sparse coverage but yielded 22 ± 4% rods compared to ∼6% in the former case. Although on a different substrate, parallels can be drawn between this work and the work of Oyama on ITO that showed that weaker nanoparticle–substrate interactions can promote anisotropic growth depending on the other growth conditions.^205^

Overall, as with the other systems discussed, if stabilizers/shape-directing reagents are not added and nucleation sites and chemical contrast are uncontrolled in the *in situ* growth process, nanostructures with poor uniformity and shape control, usually large agglomerates, result.^276^ Despite the advances made thus far in the *in situ* growth on 2D materials, the synthetic protocols developed so far rarely achieve shapes beyond spheres and rods of different sizes; the ability to access other shapes would open the door for the fabrication of novel metamaterials. For instance, nanotriangles or platelets are particularly interesting because they offer defined and quantifiable surfaces for interactions with an underlying 2D material, improving the chances of engineering the details of the interactions that govern and define the properties of the metamaterial.^278−280^

### 2.3 Patterning *In Situ*

Due to the properties described in Sections 1.2–1.4, addressable structures, such as arrays, lattices, and other complex arrangements, composed of plasmonic nanostructures have been pursued in order to explore the full potential of nanotechnology. Next, we discuss strategies focused on specifically positioning nanoparticles made with *in situ* growth and shape control into patterns.

#### 2.3.1 Physical Templates

Microfluidic devices have been used to grow nanoparticles on the internal walls of PDMS channels and SnCl_2_-sensitized spiral glass channels.^225,281,282^ Although the growth is limited to micron/millimeter scales (defined by the size of the channel), these systems demonstrate the potential for *in situ* growth to access complex three-dimensional (3D) substrates. There is interest in pursuing site-specific growth with nanometric or single-particle resolution, which can be achieved using polystyrene nanosphere self-assemblies (from nanosphere lithography) as a sacrificial mask directing *in situ* growth to exposed areas.^41,223^ However, nanosphere lithography is limited in the kinds of patterns that can be accessed, producing only hexagonal arrays with variable spacings. This limitation motivates the implementation of chemical templates, which are discussed in the next section.

#### 2.3.2 Chemical Templates

##### 2.3.2.1 Substrate-Native Chemical Templates

**Figure 10:**
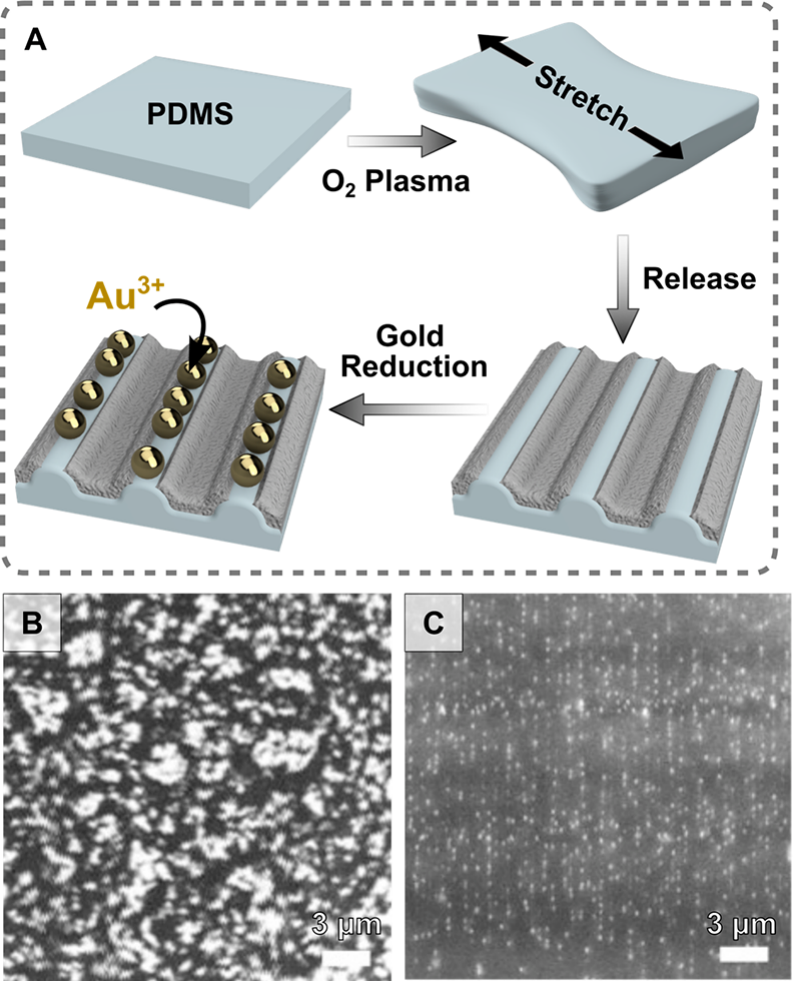
(A) Schematic showing wrinkle formation process and nanoparticle growth on polydimethylsiloxane (PDMS) “ridge cracks.” (B) Gold nanoparticles grown on PDMS without the ridge crack formation process and (C) the resulting linear arrays formed along the ridge cracks of a wrinkled PDMS substrate [adapted with permission from ref (228). Copyright 2018 Elsevier].

In chemical templates, the substrate is modified to expose/inhibit strongly reducing groups only in certain regions using patterned chemical functionalities. In some cases, the chemical specificity for growth in submicron areas can be attained using the characteristic properties of the material. In PDMS, for example, treatment with UV-ozone or oxygen/air plasma produces a brittle silica layer on the surface. This silica layer prevents exposure of the growth-active Si–H groups to the metal precursor, inhibiting growth (Figure 10A,B). Cracks in the silica layer can be formed by bending the substrate over a curved surface following oxygen-plasma treatment, or by performing the plasma activation while the substrate is stretched.^227,228^ Directional deformation of the PDMS has been used to grow ∼100 nm particle arrays on 800 nm wide and 120 nm amplitude cracks.^227^ Directional wrinkling gives a similar result, producing line arrays 400 nm wide and 30 nm deep (Figure 10C).^228^

##### 2.3.2.2 Chemical Functionalization on Generalized Substrates

**Figure 11:**
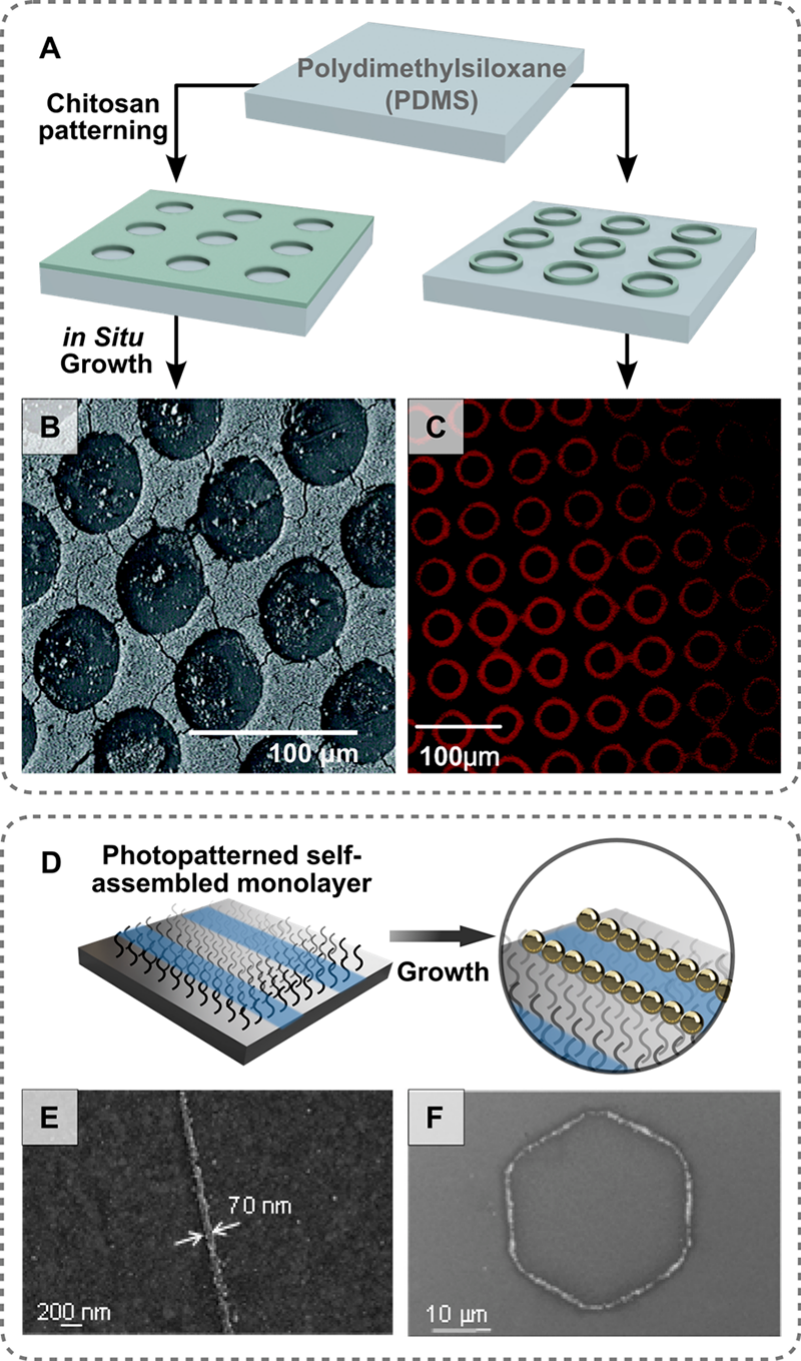
(A) Schematic showing chemical patterning with microcontact printing. (B) Scanning electron microscopy images of the inverted hole arrays following gold nanoparticle growth and (C) confocal laser scanning microscopy images of chemically patterned rings made by the same method [adapted with permission from ref (287). Copyright 2006 American Chemical Society]. (D) Schematic of photolithographically patterned chemical substrates and scanning electron microscopy images of (E) nanowires and (F) nanorings following *in situ* copper reduction [adapted with permission from ref (286). Copyright 2016 American Chemical Society].

Aside from utilizing material-specific chemical functionalities, patterning on general substrates can be achieved through the application of molecular inks that inhibit or promote growth (Figure 11). Self-assembled monolayers are often used as chemical masks and they can be patterned straightforwardly with top-down methods or by soft lithography.^219,283−286^ For instance, Wang et al. patterned a growth-active ink *via* microcontact printing (Figure 11A–C)^287^ and Ellsworth and Walker used top-down lithography to promote growth at the interface of two chemically different regions (Figure 11D–F).^286^

In general, secondary homogeneous and off-pattern nucleation are ever-present concerns. Therefore, regardless of what kind of mask is used, it is easier to control the growth using a substrate that does not promote growth in combination with a growth-positive ink. However, if it is not possible to apply a completely growth-inert material, increasing the chemical contrast between the ink and the substrate can be used to promote patterned growth (*i.e.*, kinetically favoring the reduction of the metal precursor on the ink compared to other areas). Growth-positive chitosan was patterned *via* microcontact printing on polyester, polystyrene, polyethylene, and PDMS. Chitosan accumulated into ring arrangements on topologically patterned PDMS and was also contact printed onto PDMS substrates presenting circular wells (Figure 11A–C). It was found that the nanoparticles grew selectively on areas where chitosan was present only for the PDMS substrates. This result was ascribed to the orientation of amine groups away from the hydrophobic surface. The amine groups could then attract gold ions and perform the reduction. Although the role of the Si–H moieties in the PDMS was not addressed, preferential growth appeared to occur. There are different explanations for this result: (1) PDMS presents fewer nucleation sites than chitosan-covered regions, and we are observing differences in particle density (also considering the resolution of scanning electron microscopy, SEM). (2) Hydrophilicity of the two growth-active areas, with PDMS being more hydrophobic, disfavors growth in those regions. (3) Chitosan reduces gold ions more rapidly, and continued growth of the quickly formed nuclei leads to rapid consumption of gold precursor preventing gold nanoparticle formation on the less strongly reducing Si–H groups. It is possible that all of these factors play roles, and moving forward, they should be considered for future *in situ* growth studies on PDMS-based systems.

**Figure 12:**
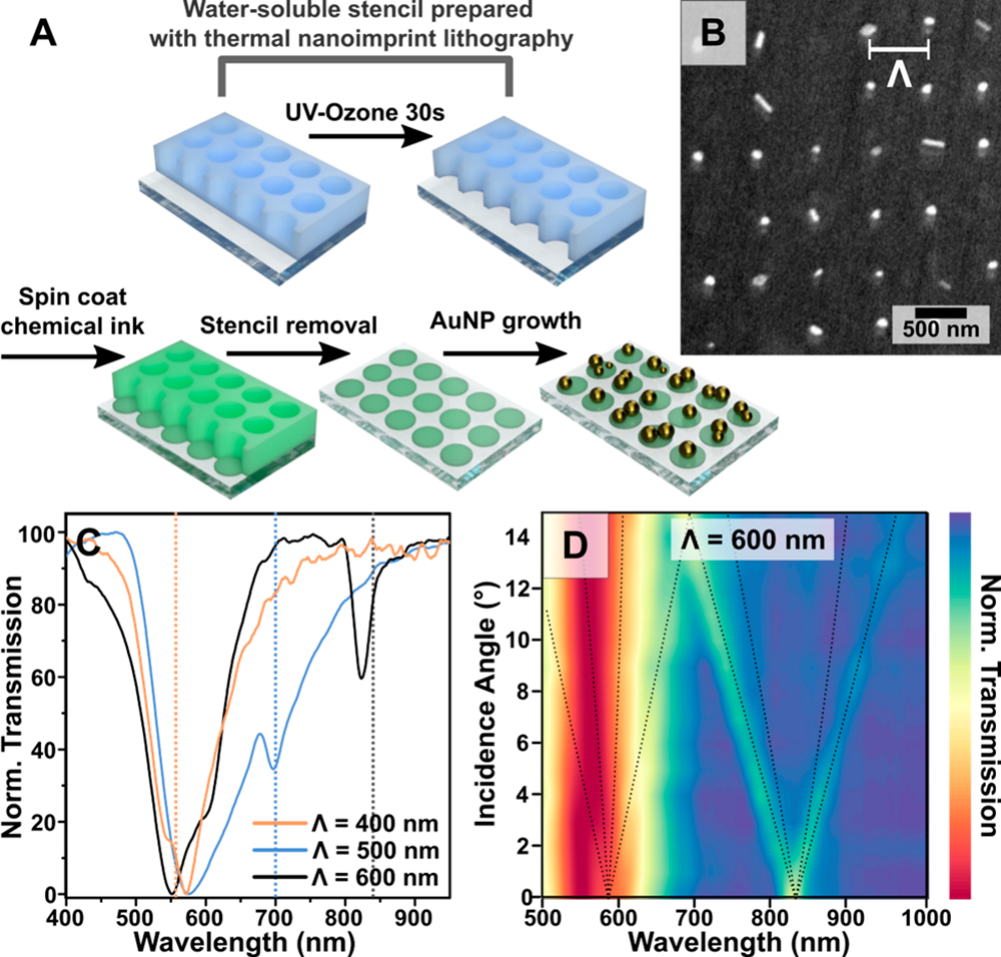
(A) Schematic of chemical patterning using water-processable polymer stencils and subsequent patterned *in situ* growth of gold nanoparticles. (B) The corresponding plasmonic arrays. (C) Transmission spectra showing the surface lattice resonances (dashed lines) for arrays with different spacings (Λ). (D) Contour plot showing the angular dependence of the surface lattice resonance peak for Λ = 600 nm. [adapted with permission from ref (288). Copyright 2022 Wiley-VCH under CC BY 4.0].

Recently, we developed a chemical patterning method applying water-soluble polymer stencils fabricated *via* thermal nanoimprint lithography (Figure 12).^288^ In this method, a growth-active ink (polymethylhydrosiloxane, a PDMS precursor) was patterned into the chemical arrays on a PDMS substrate and the chemical reduction of gold precursors was directed into those regions. One benefit of this approach was the capability to tune the geometrical parameters of the pattern (400–600 nm spacings) straightforwardly while achieving higher resolution than microcontact printing methods demonstrated previously. Patterning yields as high as 90% were attained with an average of ∼2 AuNPs (120 nm diameter) per patterned region. The main advance of this work was the observation of SLRs from the fabricated plasmonic substrates, which had not previously been realized using only the direct chemical reduction of gold ions onto chemically pattered areas (Figure 12C,D, see Section 1.4).

##### 2.3.2.3 Block Copolymer Micelle Lithography

**Figure 13:**
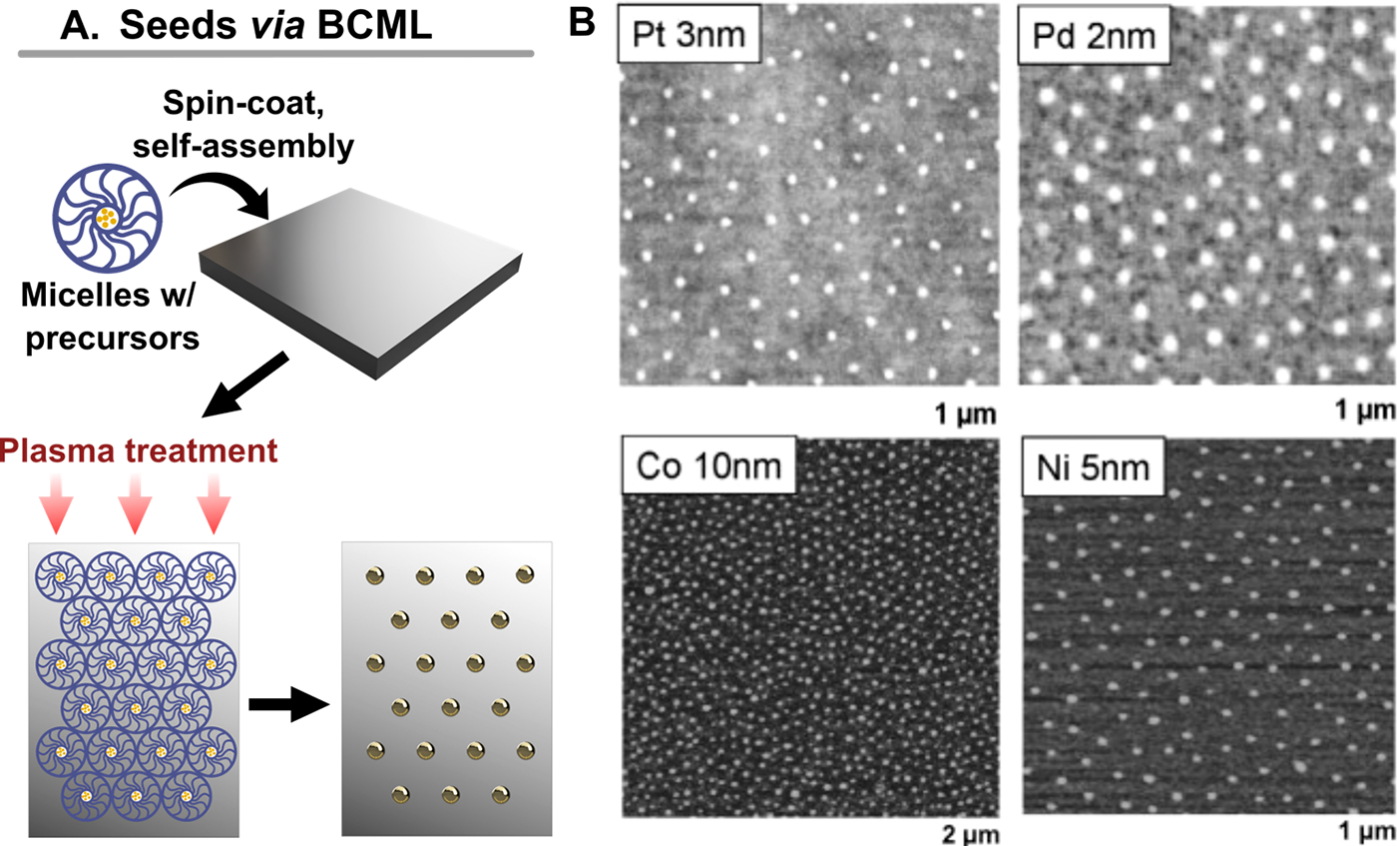
(A) Schematic of encapsulation of gold precursors in block copolymer micelles and (B) showing the self-assembly and nanoparticle formation process. (C) Images of hexagonal nanoparticle arrays synthesized from different materials using block copolymer micelle lithography (BCML) [adapted with permission from ref (301). Copyright 2006 Elsevier under CC BY-NC-ND 3.0].

One of the most popular and reliable *in situ* growth patterning methods is block copolymer micelle lithography (BCML, Figure 13).^39,289−292^ In this technique, the block copolymer micelles containing gold seed precursors (Figure 13A) are self-assembled into hexagonal arrays on the substrate *via* spin coating or dip coating onto the substrate (Figure 13B). The array spacings with this technique can be controlled by changing the assembly time, temperature, and the components of the block copolymer, while polystyrene-*b*-poly(2-vinylpyridine) and polystyrene-*b*-poly(4-vinylpyridine) are among the most commonly used, other compositions have also been explored.^293^ One limitation of standard BCML self-assembly is that only hexagonal arrays between ∼3 and 200 nm can be readily fabricated. Modifications to the technique applying coassembly of different block copolymers can create *in situ* gold nanoparticles following wave-like patterns.^39^ While recent developments by Isa expanded the technique for the preparation of square, rectangular, and Moiré patterns (performing multiple sequential BCML assemblies), the approach remains limited by presynthesis and functionalization of the nanoparticles with a specific poly(*N*-isopropylacrylamide (PNIPAm) microgel.^294−296^ Thus, accessing more complex patterns with BCML generally requires combination with top-down metal deposition,^39,297^ photolithography,^289,298^ or soft lithography.^299^ One such example is the scanning probe BCML method, which uses a cantilever to place the micelles into select regions.^300^ However, this direct-write printing method requires appropriate equipment and lengthy processing times.

### 2.4 Hybrid Patterning Approaches

There are markedly few studies that apply only *in situ* growth and provide detailed interrogation of anisotropic nanoparticle growth and/or patterning. However, the discussion in Sections 1.3 and 1.4 above indicates that there is significant motivation to use patterning to engineer the plasmonic properties of the system. Furthermore, patterning provides a means to control the number and density of nuclei on the substrate, which is beneficial for performing detailed synthetic investigations. Due to the strategic importance of this point, we include in the subsequent discussion examples beyond “*in situ* growth” that instead implement hybrid methodologies involving overgrowth of substrate-anchored colloidal seeds or seeds made by top-down methods.

#### 2.4.1 Combination of Colloidal Synthesis and *In Situ* Overgrowth

The affinity for gold nanoparticles to amine, thiol, carboxylic acid groups, *etc.* can be applied to attach colloidally prepared seeds to a substrate so that subsequent *in situ* overgrowth into larger diameters or anisotropic shapes can be performed. On oxide materials, poly-l-lysine,^302^ (3-aminopropyl)triethoxysilane (APTES),^201^ (3-aminopropyl)trimethoxysilane (APTMS), and (3-mercaptopropyl)trimethoxysilane (MPTMS)^303^ are common colloidal seed anchoring layers. We note some reports of more uniform particle substrate attachment with APTES/APTMS mixed coatings.^69^ Demonstrations of *in situ* shape control from colloidal seeds have been performed with cubes,^200^ concave structures,^199^ rods,^205^ and stars.^6,304^ The colloidal seeds can also be patterned, *e.g.*, by soft lithography.^72,219,305^ One benefit of performing *in situ* growth in this way is that it can improve the density of the overgrown nanostructures, which is useful for fabricating sensors.^199^

#### 2.4.2 *In Situ* Overgrowth of Colloidal Seeds in Microfluidic Devices

Standard colloidal syntheses can require rapid and/or simultaneous addition of reagents to the growth solution and vigorous stirring to ensure their uniform distribution.^23,128,306^ As an alternative, gold nanoparticle syntheses can be carried out in microfluidic devices with controlled reagent and thermal mixing and fluid flows.^39,282,307^ Microfluidic flow systems contain one or more channels with dimensions lower than 1 mm. Typically, the channels are fabricated with glass, silicon, or polymers, most commonly polydimethylsiloxane (PDMS or silicone), and can incorporate junctions linked with tubing made of polyetheretherketone, polytetrafluoroethylene, polyethylene, or other polymers.^308,309^ Fluid flow plays important roles in determining the degree of reagent mixing within microfluidic channels and, in the case of *in situ* growth on the substrate, determines diffusion/transport of the reagents to the surface. Reagent diffusion/mixing can be enhanced by reducing channel size^309,310^ or by modifying the device geometry to induce different flow profiles that promote reagent exchange *via* active^311^ or passive mixing.^312−315^

**Figure 14:**
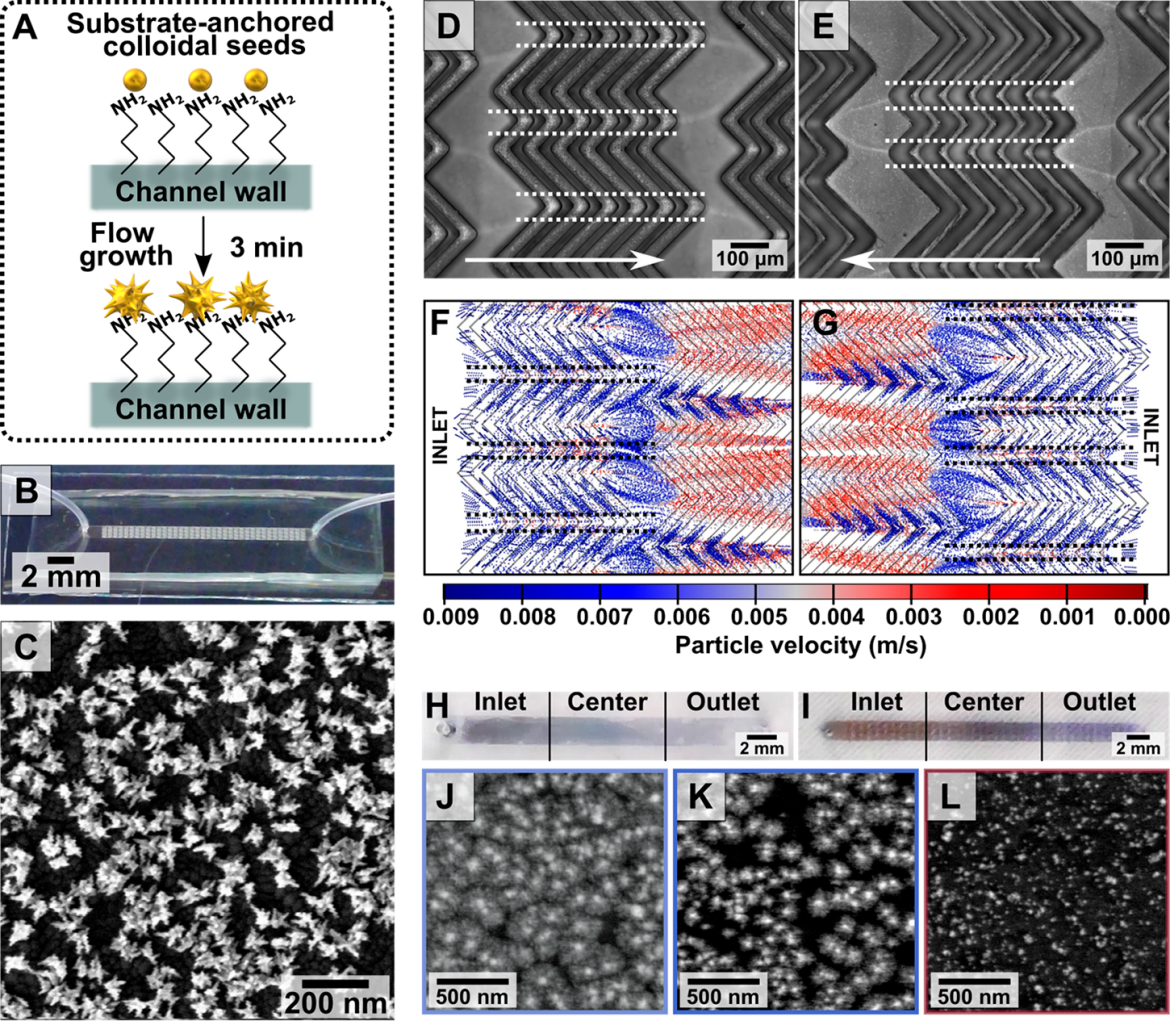
(A) Schematic showing *in situ* nanostar overgrowth in flow from a substrate functionalized with colloidally prepared seeds. (B) Digital photographs of a herringbone micromixer and (C) scanning electron microscopy image of the synthesized gold nanostars within the channel. (D, E) Brightfield microscope images of the herringbone channels after nanostar growth. The flow direction relative to the herringbone pattern is indicated with white arrows. Low-intensity areas indicated by the white dashed lines represent regions with under-developed nanostructures. (F, G) COMSOL Multiphysics particle velocity simulations which show how low intensity areas (black dashed lines) result from the fluid flow. (H, I) Digital photographs of the macroscale color gradient in the featureless and herringbone channels, respectively, caused by a depletion of growth reagents as the solution flows through the channel. (J–L) Scanning electron microscopy images showing the shape gradient along the channels with each image taken at the inlet, center, and outlet positions, respectively [Adapted with permission from ref (304). Copyright 2023 American Chemical Society].

To date, microfluidics have been primarily applied to synthesize colloidal gold nanospheres,^316^ gold-coated particles,^317^ and shaped gold particles.^314,318^ Recently, we reported *in situ* overgrowth of substrate-bound seeds. In this study, colloidal seeds were prepared and subsequently anchored to the internal walls of microreactors with different geometries. Then, the seeds were overgrown into gold nanostars by flowing a growth solution into the channel (Figure 14A). We found that flow rate, growth time, and flow profile play important roles in the *in situ* growth, affecting the development of branches.^6,304^

In this work, both “featureless” channels and “herringbone” micromixers were applied. The featureless channels are characterized by laminar flow profiles, where mass exchange occurs by diffusion, rather than advection, whereas the 3D features in the herringbone channels (Figure 14B,C) promote solution mixing. Dense gold nanostar coatings were produced on the internal walls of the channels (Figure 14C). Additionally, in the herringbone channel, different growth patterns were observed at the macroscale depending on the flow direction (Figure 14D–G). Flow velocity simulations performed with the COMSOL Multiphysics package showed that the apparent variations in nanostar development at different regions of the channel were tied to the flow profile of the micromixer. Moreover, shape gradients (going from more to less branching) were observed due to the depletion of reagents as the growth solution proceeds from the inlet to the outlet (Figure 14H–L). These initial results highlight the potential for using controlled flows to direct the growth of nanostructures on substrates, and suggest possibilities for using flows to create plasmonic substrates that contain nanoparticles with multiple morphologies and/or compositions.

#### 2.4.3 Combination of Top-down Fabrication and *In Situ* Overgrowth

**Figure 15:**
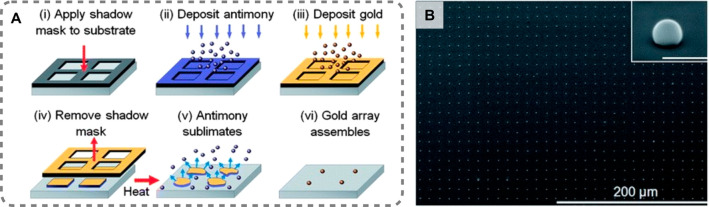
(A) Schematic of the dynamic templating procedure: (i) a shadow mask is applied to the substrate. Next, (ii) antimony is deposited to the exposed areas, (iii) followed by gold deposition. Finally, (iv) the mask is removed and (v) the sample is heated to induce gold agglomeration and antimony sublimation until (vi) the seed array is formed. (B) An electron microscopy image of the seed array prepared on a (0001)-oriented sapphire substrate. The inset shows a high magnification image of an individual seed at a 65° tilt side-view (scale bar = 1 μm) [reproduced with permission from ref (319). Copyright 2013 Royal Society of Chemistry].

With BCML and the other highlighted patterning techniques using *in situ* growth approaches, one of the main challenges is the limited tunability regarding the kinds of patterns that can be fabricated (see section 2.3.2.3). The Neretina group addressed this limitation by taking hybrid approaches, such as using nanoimprint lithography (NIL) or top-down lithography, which access different arbitrary patterns.^196,202,218,319^ Top-down dynamic metal templating and NIL were combined to create highly tunable and reproducible arrays of gold seeds on which synthetic shape-control studies could be performed: First, NIL or a lithographically prepared shadow mask is used as a physical mask (Figure 15). Subsequent deposition of Sb, then Au onto the polymer hole array is performed, the mask is lifted off, and the Sb–Au disks are heated to sublimate the Sb, creating gold nanoparticle seeds.

### 2.5 Stabilization of *In Situ*-Grown Nanoparticles

In the context of colloidal gold nanoparticles, the idea of stabilization calls to mind the use of a capping ligand or surfactant to sustain the colloidal stability of NPs in suspension through steric and/or Coulombic repulsion.^320^ For substrate-grown NPs, one no longer needs to consider colloidal stability. This difference is one of the most interesting benefits of such growth schemes, making surfactant-free NPs^321^ for applications like heterogeneous catalysis and sensing, or for producing nanostructures amenable to later functionalization. However, in some instances, especially when patterning is involved, some reports emphasize the importance of “stabilizing” the particles to remain in their predetermined position on the surface. In the work of Landeke-Wilsmark et al. on the *in situ* synthesis of 10–50 nm gold nanospheres from seeds prepared with BCML, they suggested that stabilizing ligands may even be detrimental for *in situ* growth if the interaction between the nanoparticle and the substrate is too weak.^217^ In fact, when performing the BCML seed fabrication and sphere overgrowth on silicon substrates on which gold nanoparticles weakly adhere, the presence of citrate led to gold nanoparticle detachment. They also found that the particles on the substrate can be secured in place with a single atomic layer deposition cycle of HfO_2_ and Al_2_O_3_ on the seeds prior to overgrowth. In the earlier synthesis of spheres 50–150 nm in diameter by Löhmuller and co-workers, nanoparticles were fixed in place on the surface *via* assembly of a lipid bilayer membranes.^322^

### 2.6 Bottom-up Size and Shape Control

Next, we describe some examples of anisotropic growth *in situ,* including those that use patterning (*i.e.*, BCML),^197,198,217^ nonpatterned *in situ* growth,^195,323^ and *in situ* overgrowth on seeds prepared from colloidal solution.^199,204,205^ Thus far, examples of shapes accessible with *in situ* shape control include spheres of different sizes,^217,324^ polyhedra/triangular plates,^79,198,202,325^ branched structures,^6,197,203,218,288^ nanorods,^201,205,206,326,327^ concave structures,^199^ and standing nanowires^204,328^ (some examples are highlighted in Table 1). In the next sections, we discuss selected work focused on the syntheses of various anisotropic structures *in situ*, beginning with nanorods, then continuing to platelets, stars, and nanowires.

**Table 1:** Summary Highlighting Different Nanoparticle Sizes and Shapes Prepared by *In Situ* Overgrowth in Combination with *In Situ* Seeding or Other Methods (As Indicated)^a^

Particle type	Size (nm)	Seeding method	Substrate	Ref
Spheres	7–several hundred	*In situ* growth	PDMS (bulk and microfluidic channels)	(238, 329, 330)
	N/A	*In situ* growth	PNIPAM	(216)
	2–25	*In situ* growth	Graphene/graphene oxide	(254−257, 276)
	10–10s	*In situ* growth	WSe_2_/MoS_2_	(71, 249, 265)
Rods	100–600 (*l*) × 10–30 (*w*)	Immobilized colloidal seeds	ITO/mica/silicon	(68, 205, 326)
	2,200–3,500 (*l*) × ∼100 (*w*)	Immobilized colloidal seeds	Graphene	(277)
Aligned rods	600 (*l*) × 30 (*w*);	Immobilized colloidal seeds	Silicon	(201)
	200 (*l*) × 20 (*w*); 450 (*l*) × 40 (*w*)	Immobilized colloidal seeds	PMMA	(206, 327)
Nanospikes	50–400	*In situ* growth	Carbon nanofiber scaffolds	(323)
Nanostars	300–400	*In situ* growth	PDMS	(59)
	∼125	Immobilized colloidal seeds	Glass/ITO (slides and microfluidic channels)	(6, 304)
Concave cubes	70–140	Immobilized colloidal seeds	Paper/cellulose	(331)
Nanowires	6–17 (*d*)	Immobilized colloidal seeds	Glass	(204)
	10–30 (*d*)	*In situ* growth (from Pd seeds)	Polycarbonate foil/carbon fiber cloth	(328)
Ag@Au shells	Several hundred	*In situ* growth	PDMS	(224)
Sphere arrays/patterns	40–100	*In situ* growth + tNIL	PDMS	(288)
	1–60	BCML	Glass/silicon	(203, 217, 322, 324)
	∼100	*In situ* growth + μCP	PDMS	(287)
	∼100	Crack templated lithography	PDMS	(227, 228)
Nanostar arrays	∼100	*In situ* growth + tNIL	PDMS	(288)
	∼10–70	BCML	Glass	(197, 203, 332)
	65–240	Dynamic templating	Sapphire	(218)
Triangle/platelet arrays	40–60	BCML	Silicon nitride membrane	(198)
	200–400	Dynamic templating	Sapphire	(79, 202, 202)
Multimetallic nanocluster arrays	∼90	SPBCML	Silicon nitride membrane	(200, 300)
Chiral arrays	25 (*h*); several hundred (*d*)	Dynamic templating	Sapphire	(333)
Cage arrays	∼200	Dynamic templating	Sapphire	(334)

a t-NIL = thermal nanoimprint lithography; (SP)BCML = (scanning probe) block copolymer micelle lithography; μCP = microcontact printing; PDMS = polydimethylsiloxane; PNIPAM = poly(*N*-Isopropylacrylamide); PMMA = poly(methyl methacrylate); ITO = indium tin oxide.

#### 2.6.1 Nanorods

**Figure 16:**
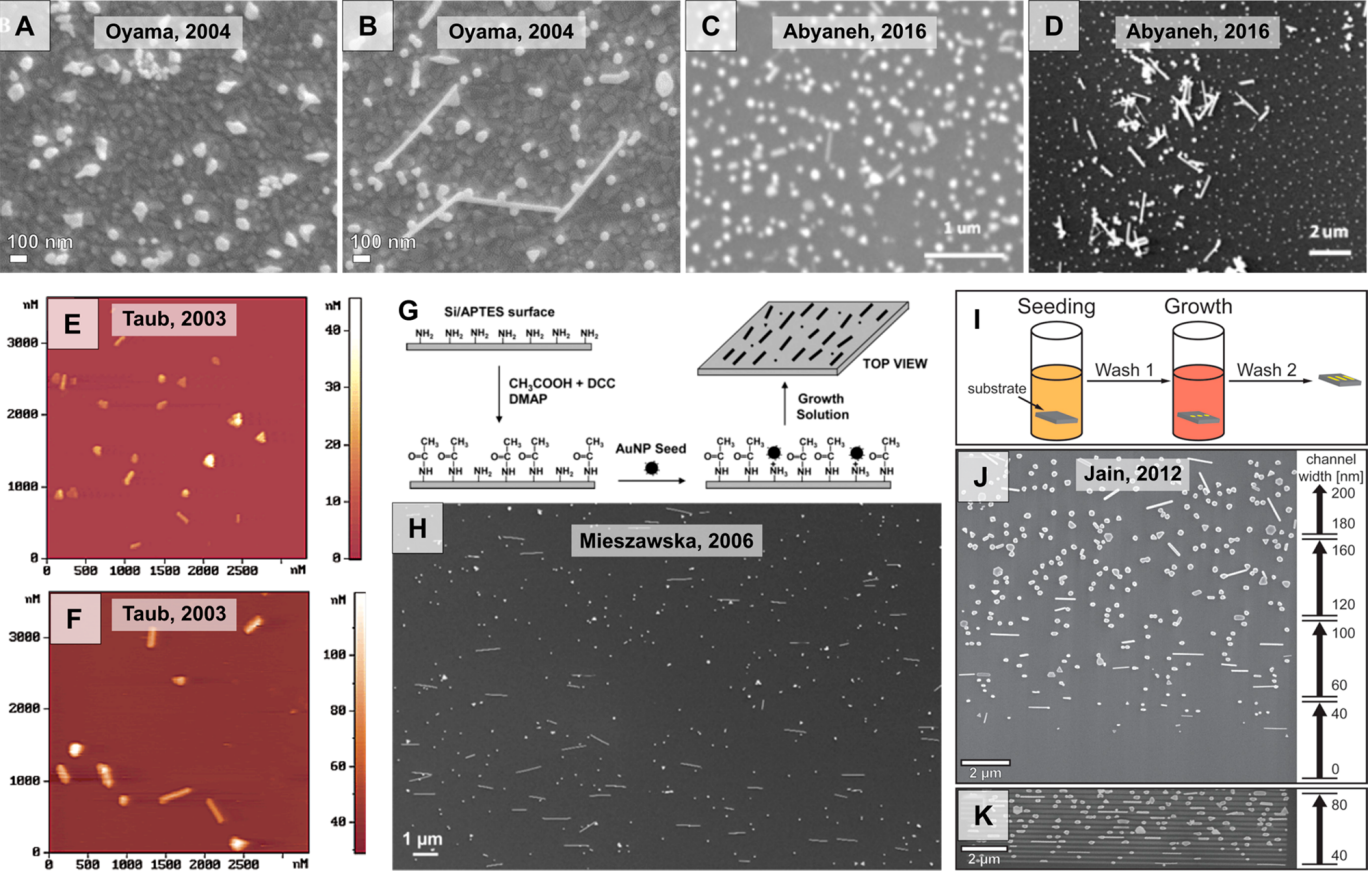
Scanning electron microscopy (SEM) images of gold nanoparticles prepared by Oyama with the growth recipe of Murphy on indium tin oxide functionalized with (A) thiol and (B) amine groups [adapted with permission from ref (205). Nanorod SEM images by Abyaneh on (C) high- and (D) low-molecular weight polymethylmethacrylate substrates [adapted with permission from ref (206). Atomic force microscopy images of nanorods obtained by Taub after (E) one growth step and (F) three rounds of growth [adapted with permission from ref (326). Copyright 2003 American Chemical Society]. (G) Schematic of the surface amidation step and aligned nanorod growth on Si(100) substrates by Mieszawska. (H) SEM image of the aligned rods [adapted with permission from ref (201). Copyright 2006 American Chemical Society]. (I) Schematic of the growth procedure for aligned rods on a Si substrate patterned with PMMA nanochannels prepared *via* electron beam lithography. (J, K) Scanning electron micrographs of the aligned rods in channels with different dimensions [adapted with permission from ref (327). Copyright 2012 American Chemical Society].

Similar to colloidal synthesis, the chemical environment of the growth can be modified to tune the kinetics of the reaction to favor the formation of thermodynamically favored anisotropic products, such as gold nanorods.^127,129,335^ Gold nanorods are popular anisotropic particles used for thermoplasmonic applications and sensing.^96,336^ Colloidal synthesis recipes are often used as starting points for the synthesis of different shapes *in situ*. In the colloidal synthesis method by Murphy, CTAB and AgNO_3_ are used as shape-directing reagents.^130^ However, certain factors differentiate bottom-up synthesis on substrates and in colloidal solutions. For example, surface-nuclei or surface-particle interactions can affect anisotropic growth. The Oyama group, for one, explored nanorod growth from colloidal 4 nm citrate-capped seeds attached to ITO substrates with APTES and MPTMS (Figure 16A,B).^205^ The substrates were immersed in an overgrowth solution containing the shape-directing reagents over 24 h, producing high aspect ratio nanorods but with rather low yield. When seeds were more strongly bound to the substrate with MPTMS, fewer rods were produced compared to seeds bound weakly with APTES. The substrate also affected growth of nanorods on polymethylmethacrylate (PMMA) by Abyaneh and co-workers, with low molecular weight PMMA yielding more rods; however, the mechanism behind this result was not determined (Figure 16C,D).^206^

In colloidal nanorod syntheses, it is common to apply multiple growth steps to increase the aspect ratio of the nanostructures.^129,130,335^ Multiple growth steps are facile to apply *in situ* since growth can be stopped by simply removing the growth solution from the substrate. Taub and colleagues explored implementation of multiple *in situ* growth steps for nanorods, where citrate seeds 3.5 nm in diameter were anchored to mica substrates with APTMS.^326^ Applying synthetic parameters from the group of Murphy,^337^ the growth time was varied to optimize the yield of structures with aspect ratio >2, giving final yields of 15% after multiple steps, where with only one step, the obtained shape yield was only 4% (Figure 16E,F). Overall, the limited improvement in yield was attributed in part to the affinity for CTAB for the mica substrate, preventing gold precursor from reaching the surface-bound seeds. Despite the nearly 4× improvement in yield of high aspect ratio rods, a disadvantage of *in situ* growth was identified: in colloidal syntheses, centrifugation can be used to isolate certain products and improve overall yield (as is common for rods^130,337^ and triangles^338^). For substrate-anchored products, it is *not* possible to isolate the desired shapes in this way, hence the relatively low yields in the majority of reports included in Figure 16. It was also postulated that the seeds may have developed fewer twin defects caused by binding to the substrate, although, because the seeds were prepared colloidally, this explanation is unlikely. Later in our analysis of *in situ* platelet syntheses, we elaborate on the importance of seed/particle defect orientation relative to the substrate.

Mieszawska et al.’s work highlighted the importance of substrate-particle interactions, demonstrating that amide-functionalized Si(100) substrates could be used to grow rods aligned in the same direction (Figure 16G,H).^201^ Although the mechanism for aligned growth is not understood, aligned rods were not observed on glass or on Si(100) functionalized with amine or thiol groups, suggesting that both the substrate crystal structure and functionalization play roles influencing particle orientation. Aligned rods can also be prepared with the assistance of top-down fabrication of PMMA nanochannels on Si substrates (Figure 16I–K).^327^ Lastly, another observation is that for all of the above-mentioned syntheses, while there are reports of synthesized rods aligned in the same direction with their longitudinal axis parallel to the substrate, no standing nanorods were formed.

#### 2.6.2 Triangles and Other Platelets

**Figure 17:**
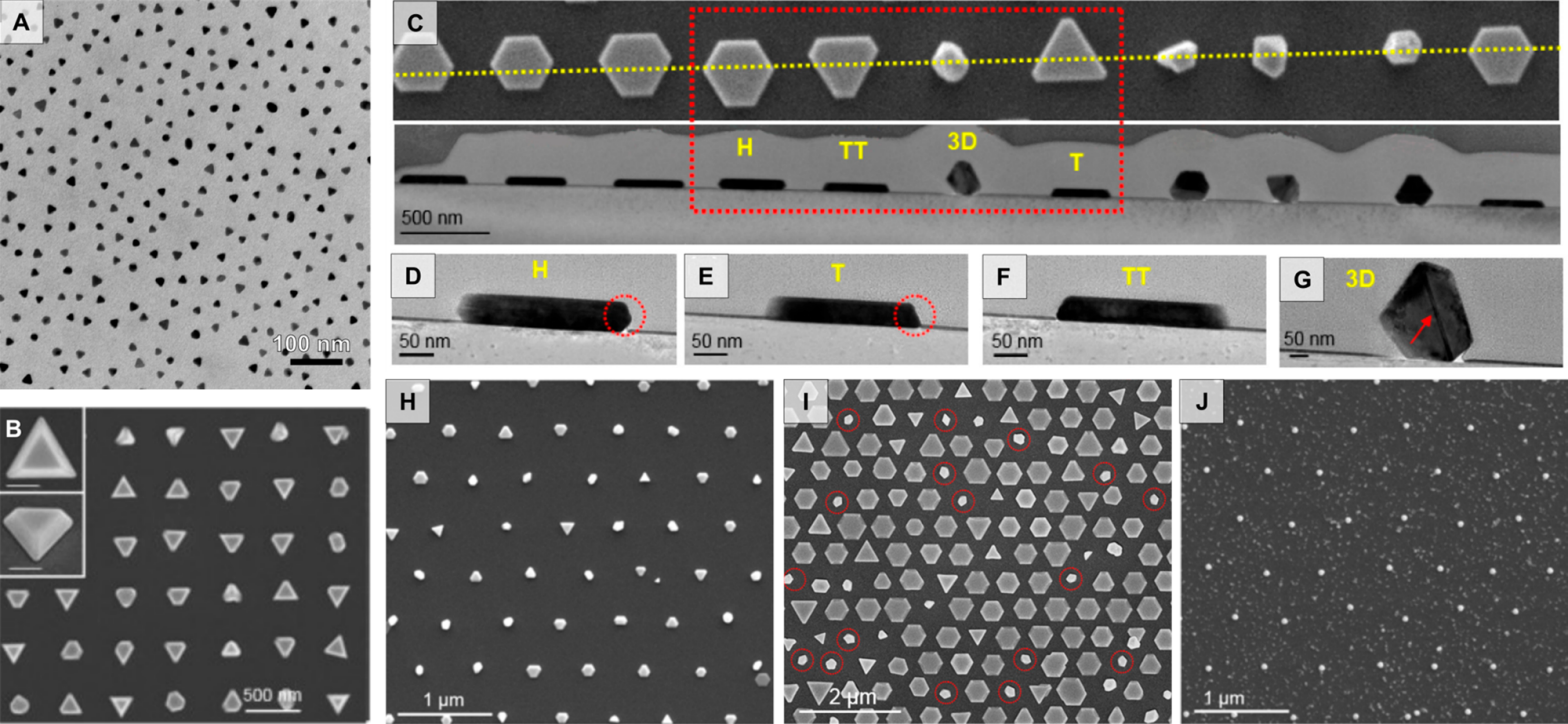
(A, B) Nanotriangles synthesized by (A) Kumar et al. [adapted from ref (198) with permission. Copyright 2007 American Chemical Society] and (B) Preston et al. [adapted with permission from ref (340) Copyright 2019 Elsevier]. (C) (*Top*) Scanning electron microscopy image of particles and (*bottom*) cross-sectional transmission electron microscopy (TEM) images of nanoplates. (D) Higher magnification cross-section TEM images of (D) hexagonal (H), (E) triangular (T), (F) truncated triangular (TT), and (G) three-dimensional (3D) morphologies. (H–J) Scanning electron micrographs of nanoplates grown at (H) pH = 2.0, (I) 3.0, and (J) 5.. [adapted with permission from ref (202). Copyright 2019 American Chemical Society].

Kumar and co-workers presented good yield of nanotriangles *in situ* (∼70%) from overgrowth of 3–4 nm seeds fabricated in hexagonal arrays with BCML.^339^ They found that applying synthetic conditions reported by Sau and Murphy that mainly produced “square” particles (cubes or tetrahedra) in colloidal conditions instead mainly yielded triangular shapes with edge lenghts of 40–60 nm (Figure 17A). The authors suggest that the difference in results is due to the structure of the seeds prepared by BCML presenting different defects than those prepared in solution. Furthermore, diffusion of reagents to substrate-bound seeds may have an effect. As with the nanorods, the nanostructures presented here were oriented with their high-surface area faces parallel to the substrate. Another disadvantage of *in situ* growth is that, in many cases, SEM characterization must be used in place of transmission electron microscopy (TEM), which typically provides better resolution. In the case of *in situ* growth on nonconductive substrates, characterization by electron microscopy becomes even more difficult. Kumar innovatively grew nanostructures directly on TEM grids coated with a thin Si_3_N_4_ membrane so that the shapes of the particles could be evaluated straightforwardly.^339^

Neretina’s group used dynamic templating to fabricate seeds for the epitaxial overgrowth of nanotriangles (Figure 17B) and *in situ* plasmon-assisted growth of hexagonal nanoplate arrays (Figure 17C–J). The seeds were predominantly oriented with the [111] axis normal to the surface, with stacking defects parallel to the substrate.^202^ The seed orientation was a result of epitaxial formation on the underlying sapphire substrate. In addition, the yield of platelets *in situ* was significantly higher than in solution (78% *vs* 21%, respectively). Here, TEM analyses were performed on samples prepared by focused ion beam cross-sectioning, discovering that seeds containing defects supporting platelet growth oriented perpendicular to the substrate surface only produced truncated 3D structures. In fact, as with triangles and rods, no standing or nonsubstrate parallel platelets were observed, supporting the idea that horizontal growth is promoted *in situ*. Furthermore, as with colloidal systems, pH can affect the shape-yield, with low pH giving more triangular platelets and higher pH giving poorly defined geometries. This observation suggests that the kinetics of growth are important in controlling *in situ* overgrowth into anisotropic shapes, because polyvinylpyrrolidone (PVP) becomes a weaker capping agent at higher pH, causing growth to occur more quickly. As described in the previous section, centrifugation cannot be used to select for the desired products in a substrate growth format. However, Neretina’s group recently applied sonication to remove byproducts from the platelet synthesis, improving the yield from 78% to 95%.^325^ Thus, this sonication approach can potentially serve as an analogue to purification *via* centrifugation for *in situ* systems.

#### 2.6.3 Branched Structures

**Figure 18:**
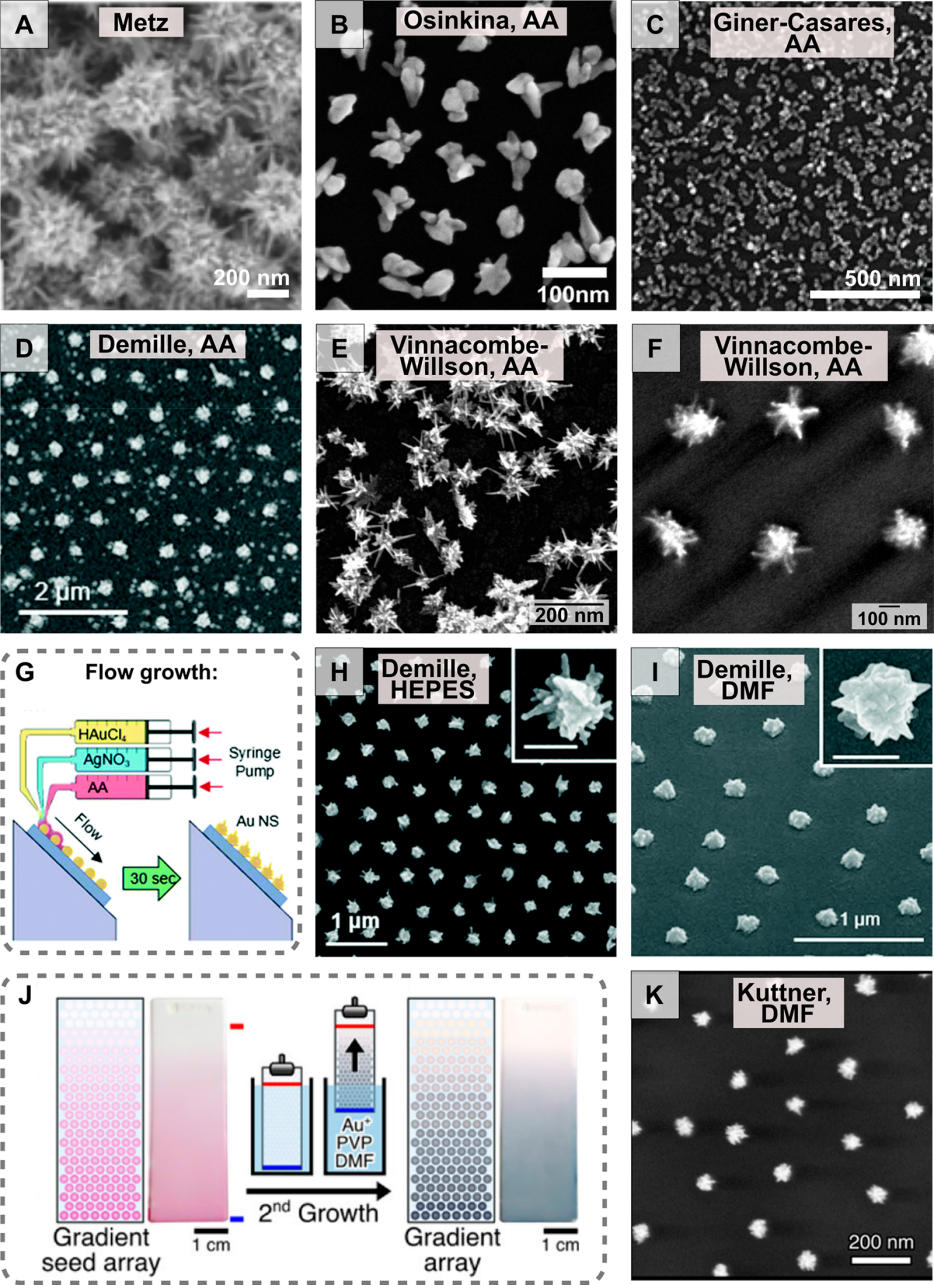
(A–F) Scanning electron micrographs of branched nanostructures prepared in various ways [adapted with permission from references (A) (323) (Copyright 2006 American Chemical Society), (B) (197) (Copyright 2013 American Chemical Society), (C) (62) (Copyright 2016 Wiley VCH under CC BY-NC-ND 4.0), (D, H, I) (218) (Copyright 2020 Royal Society of Chemistry), (E) (6) (Copyright 2020 American Chemical Society under NC-BY-NC-ND 4.0), (F) (288) (Copyright 2022 Wiley VCH under CC BY 4.0), and (K) (203) (Copyright 2021 American Chemical Society)]. (G) Schematic of flow growth performed by the group of Neretina [adapted from ref (218). Copyright 2020 Royal Society of Chemistry]. (J) Schematic of nanostars grown using seed size gradients [adapted from ref (203). Copyright 2021 American Chemical Society]. AA = l-ascorbic acid; HEPES = 4-(2-hydroxyethyl)-1-piperazineethanesulfonic acid; DMF = dimethylformamide.

Kinetically favored branched gold nanostructures were synthesized *in situ* in the mid-2000s,^323^ just as early colloidal nanostar syntheses were being developed.^119,148^ Metz et al. prepared gold spikes on vertically aligned silver-coated carbon nanotubes (Figure 18A).^323^ More recent demonstrations of *in situ* growth of branched nanostructures take inspiration from colloidal recipes, applying growth solutions based on established protocols. For example, overgrowth into branched structures driven by reducing agent ascorbic acid has been explored, combining AA with shape directing agents like AgNO_3_ and CTAB (Figure 18B–F).^6,197,218,288,332^*In situ* growth based on Xie’s protocol^119^ applying 4-(2-hydroxyethyl)-1-piperazineethanesulfonic acid (HEPES) and AgNO_3_, has also been demonstrated (Figure 18H). Demille and Kuttner (Figure 18D–I) both established *in situ* overgrowth using *in situ* hybrid methods (dynamic templating and BCML, respectively) of nanostars using a recipe based on the PVP/DMF colloidal synthesis of Kumar.^148^

In the work of Demille, secondary nucleation in the growth solution away from the substrate appeared to inhibit anisotropic growth *in situ*.^218^ Even though the system was designed with anchored nuclei to promote growth on the substrate selectively, the competition between the surface-bound nuclei and colloidal nuclei for growth reagents persists as a barrier for controlled *in situ* growth, which has also been noted elsewhere.^217^ It is expected then that the DMF-driven synthesis was the most successful, since it produces less secondary nucleation in the same amount of time as the more rapid AA- and HEPES-driven syntheses. Expanding upon the role of secondary nucleation, “flowing” the growth solution over the substrate produces branched structures in the AA-driven case, where overgrowth in stationary solution did not (Figure 18G). The authors attribute this contrast to differences caused by the no-slip condition, similar to the rationalization regarding reagent diffusion suggested in the rod synthesis by Taub^326^ and the nanotriangle synthesis by Kumar.^198^ However, between stationary solution and the applied flowing solution, mass transfer should still be dominated by diffusion rather than convection.^6,309^ Another possible contribution to this result could be that flowing the growth solution replenishes freshly mixed reagents for the substrate-bound seeds, with flow carrying away or removing colloidal nuclei throughout the synthesis, reducing the competition between colloidal and surface growth. Regardless, even for flow growth, the negative effects of secondary nucleation can still be observed *via* the population of particles outside of the areas containing seeds (Figure 18D). Similarly, looking back at the work on platelets by the same group, an increase in the presence of particles outside the patterned regions (likely from secondary nucleation) also coincided with a decrease in yield of the target products (Figure 17J).

**Figure 19:**
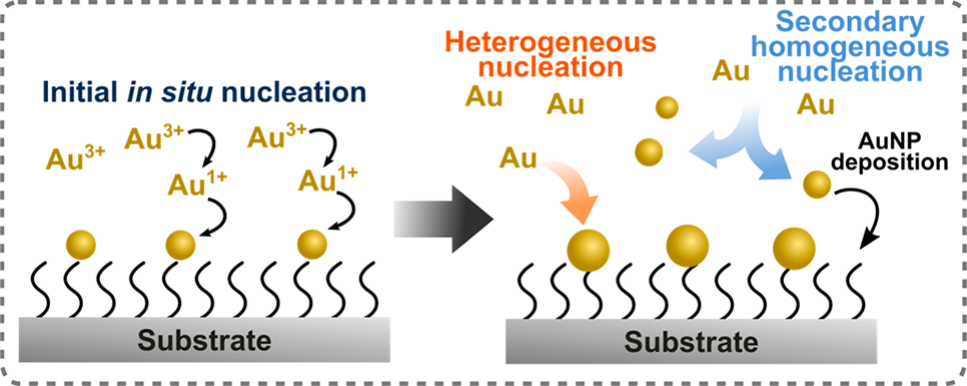
Schematic depicting heterogeneous and secondary nucleation in *in situ* growth systems that incorporate strong reductants on the substrate [adapted with permission from ref (288). Copyright 2022 Wiley VCH under CC BY 4.0].

These results are evidence that, while the promotion of nuclei formation only on the substrate either by *in situ* (surface functionalization with strong reductants) or hybrid methods assists in significantly limiting secondary nucleation (illustrated in Figure 19), it does not guarantee the complete avoidance of secondary nucleation, especially in the synthesis of kinetically favored products. This idea was central to our work directly addressing secondary nucleation by adding HCl, modulating surfactant concentration and identity, and optimizing the water source to enhance the production of branched structures (Figure 18E,F).^6^

#### 2.6.4 Vertical Growth: Nanowires

Although shape control *in situ* is significantly less studied than colloidal synthesis, there is a great opportunity for synthesizing shape-controlled plasmonic structures and even fabricating plasmonic substrates that are not routinely produced with colloidal self-assembly. While side-to-side or end-to-end assembly of elongated nanostructures can be performed colloidally,^341,342^ and vertically aligned assemblies can be prepared at high packing-fraction,^77,343−345^ one benefit of *in situ* growth is the possibility of synthesizing standing nanorod or wire monolayers with extremely high yield and tunable packing fraction. The synthetic studies of these structures provide interesting insights into the mechanistic differences of *in situ* growth compared to colloidal systems.

**Figure 20:**
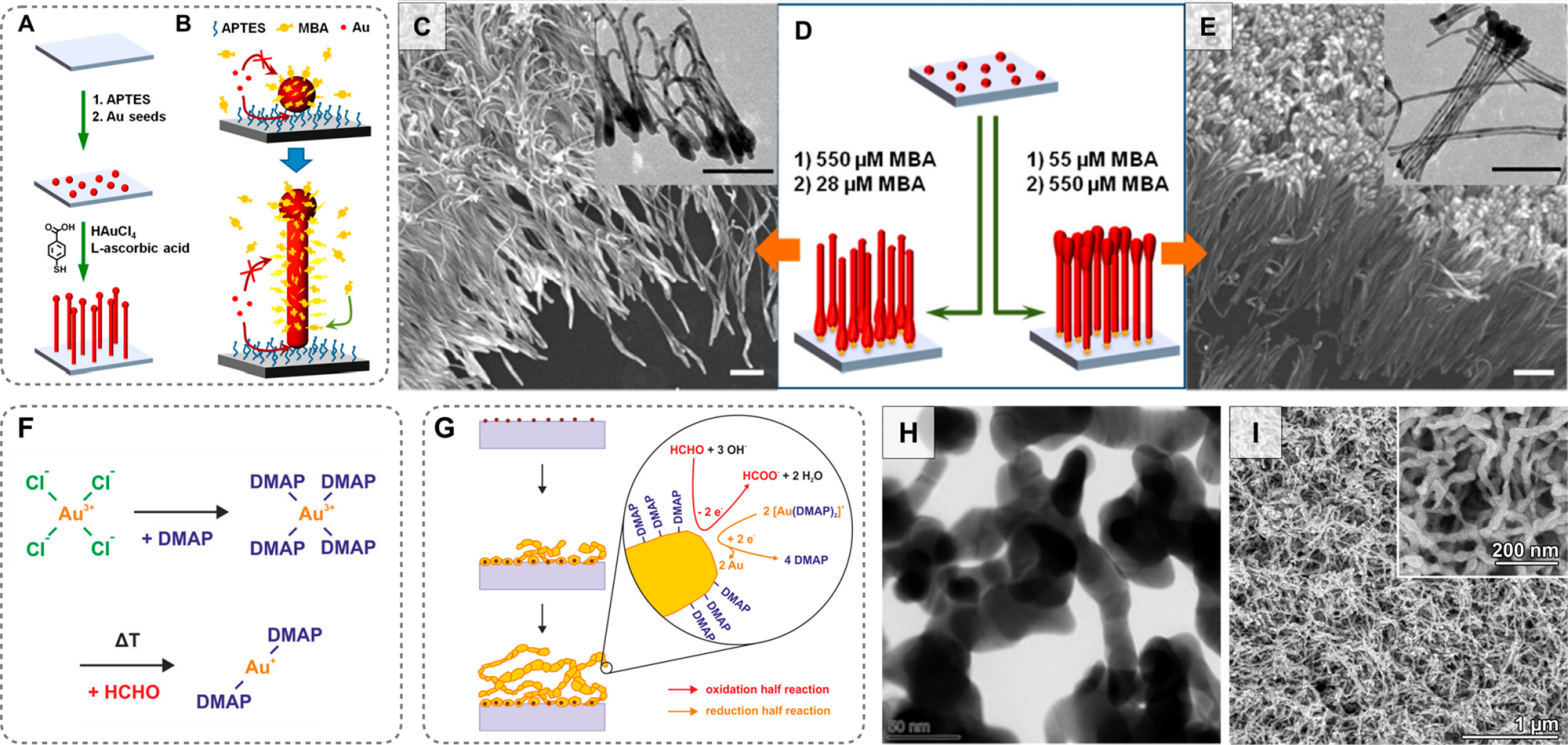
(A) Schematic showing colloidal seed attachment to glass with (3-aminopropyl)triethoxysilane (APTES) and subsequent growth into nanowires with mercaptobenzoic acid (MBA), ascorbic acid, and gold salt. (B) Depiction of the fast passivation of MBA at the nanowire sides and selective growth *via* attachment of gold atoms at the substrate interface. (C–E) Scanning electron micrograph showing the result of decreasing wire width with (C) decreased and (E) increased MBA concentration in the latter growth steps as shown in the schematic in panel (D) [adapted with permission from ref (204). Copyright 2013 American Chemical Society]. (F) Reactions occurring in the growth solution and (G) schematic of Au nanowire growth by Muench et al.: Pd seeds were made by electroless plating, then a solution containing 4-dimethylaminopyridine (DMAP) as a capping ligand, gold salt, and formaldehyde as a mild reducing agent is added to produce nanowires with high yield. As depicted in panel G, continuous growth occurs at the wire tips. (H, I) Electron microscopy images of the products [adapted with permission from ref (328). Copyright 2017 American Chemical Society under CC BY-NC-ND].

There are certain conditions that can preferentially promote vertical growth from the substrate, showing that *in situ* growth provides opportunities for controlling the orientation of the particles. He and co-workers were able to synthesize standing nanowires in high yield (Figure 20A–E).^204^ They proposed that the vertical growth is due to the presence of strongly binding thiol ligands (mercaptobenzoic acid, MBA) that only permit addition of the gold atoms at the interface between the seed and the substrate, where the ligand shell is most disordered (Figure 20A,B). The sides and tips of the nanowires, on the other hand, are quickly passivated by MBA, making it unfavorable for gold atoms to be added in those regions. The role of the density of the ligand was supported by the execution of the synthesis with different ligand concentrations during growth. They found that the wires had thicker tails and thinner heads when the MBA concentration was reduced during the synthesis. Conversely, when the ligand concentration was increased, thinner heads and thicker tails were produced (Figure 20C–E). In a later study, gold nanowires were instead grown by promoting growth selectively at the nanowire tips, as shown in Figure 20F and G.^328^ The wires exhibit curves and changes in diameter along the longitudinal direction, which are proposed to result from changes in the growth direction as addition of gold atoms is occurring at the wire tips (Figure 20H,I). For further details see recent reviews on the subject.^79,195^ Various groups have also demonstrated standing structures with other metals, such as silver platelets.^321,346,347^ Overall, these results show that the substrate can be used to influence the direction of growth, an advantageous feature of *in situ* methods.

Beyond metals, standing semiconductor nanowires can be formed at lower temperatures *via* the presence of a liquid alloy catalyst and various techniques based on this idea have been developed (vapor–liquid–solid, solution-liquid–solid, supercritical fluid–liquid–solid, *etc.*).^348,349^ While semiconductor nanowire growth differs significantly from *in situ* noble metal nanoparticle growth and is normally self-seeded (wires grown of the same material as the substrate) or catalyzed by gold nanostructures created *via* metal evaporation and sputtering, one can envision potential synergies between these techniques. Therefore, we direct interested readers to recent comprehensive reviews on the topic.^348−350^

#### 2.6.5 Other Shapes

**Figure 21:**
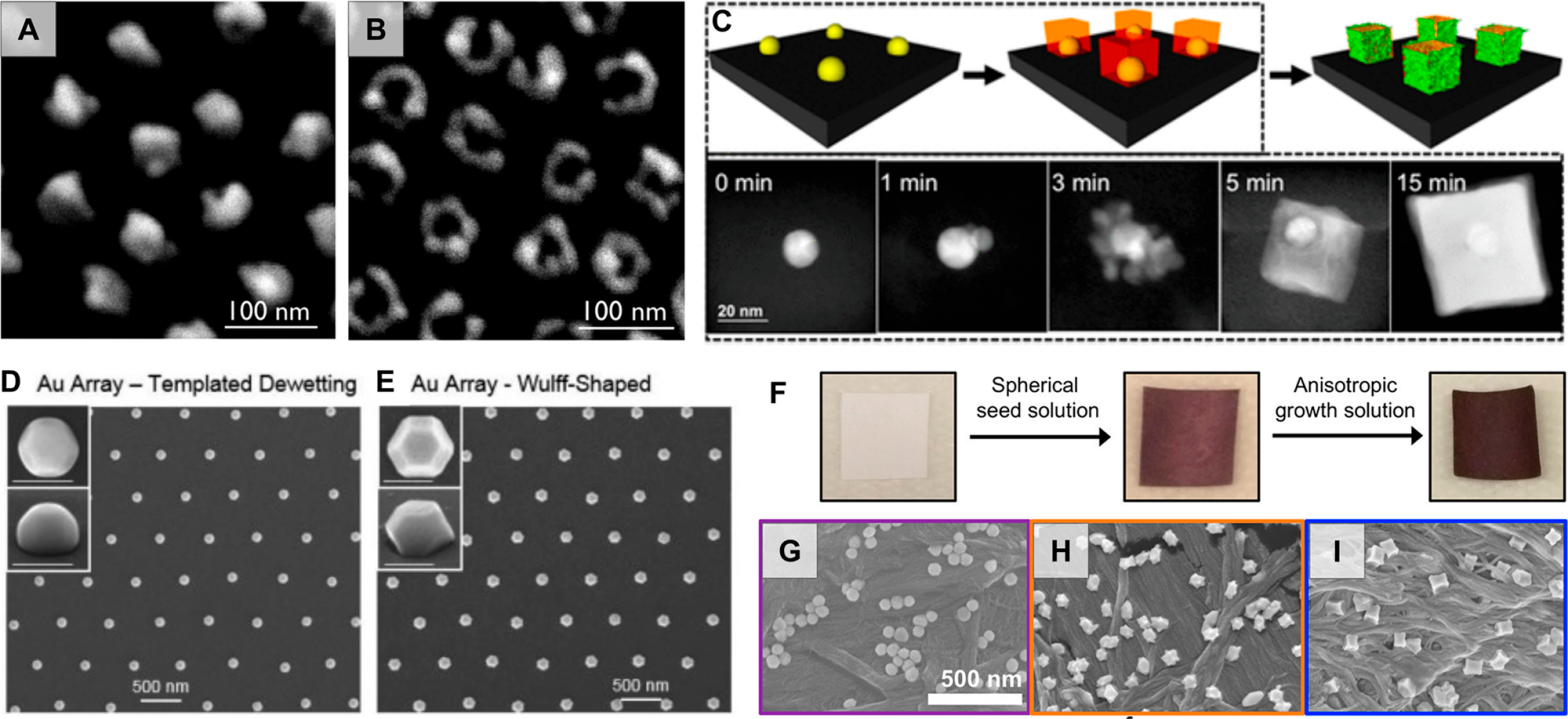
Scanning electron microscopy (SEM) images of (A) nanopotatoids and (B) nanorings fabricated by block copolymer micelle lithography and *in situ* photoreduction of gold precursors [adapted with permission from ref (351). Copyright 2015 Royal Society of Chemistry]. (C) Schematic of gold seed arrays prepared by scanning probe block copolymer micelle lithography and overgrowth into a multimetallic (Au@Pd@Pt core–shell-dendrite nanoparticles) cube (*top*) and low-angle annular dark-field scanning transmission electron microscopy images of Au@Pd core–shell nanoparticles at different growth times (*bottom*) [adapted with permission from ref (200). Copyright 2015 American Chemical Society]. (D) Dewetted and (E) Wulff-shaped truncated gold nanoparticle SEM images [adapted with permission from ref (340). Copyright 2019 Elsevier]. (F) Photographs of paper after attachment of colloidal seeds (*middle*) and following overgrowth into concave cubes (*right*). (G–I) SEM images of the paper substrates (G) with seeds, (H) after overgrowth into concave rhombic dodecahedra, and (I) concave cubes [adapted with permission from ref (199). Copyright 2018 American Chemical Society].

The BCML method has also been used to synthesize anisotropic “nanopotatoids” and nanoring arrays (Figure 21A,B).^351^ Although BCML relies on photoreduction rather than chemical reduction, this example demonstrates the potential versatility of substrate-bound anisotropic structures that could be targeted with *in situ* growth. Scanning probe BCML is similar to BCML, but uses a cantilever to print block copolymer micelles containing metal precursors onto the substrate.^200,300,352^ Recent scanning probe BCML studies measure the positions of the seed particles relative to the substrate following overgrowth. Specifically, overgrowth was performed with different metals, including gold, silver, platinum, and palladium. Due to the contrast of the different overgrowth and seed metals, HAADF-STEM revealed that the seed lay asymmetrically close to the substrate (Figure 21C).

**Figure 22:**
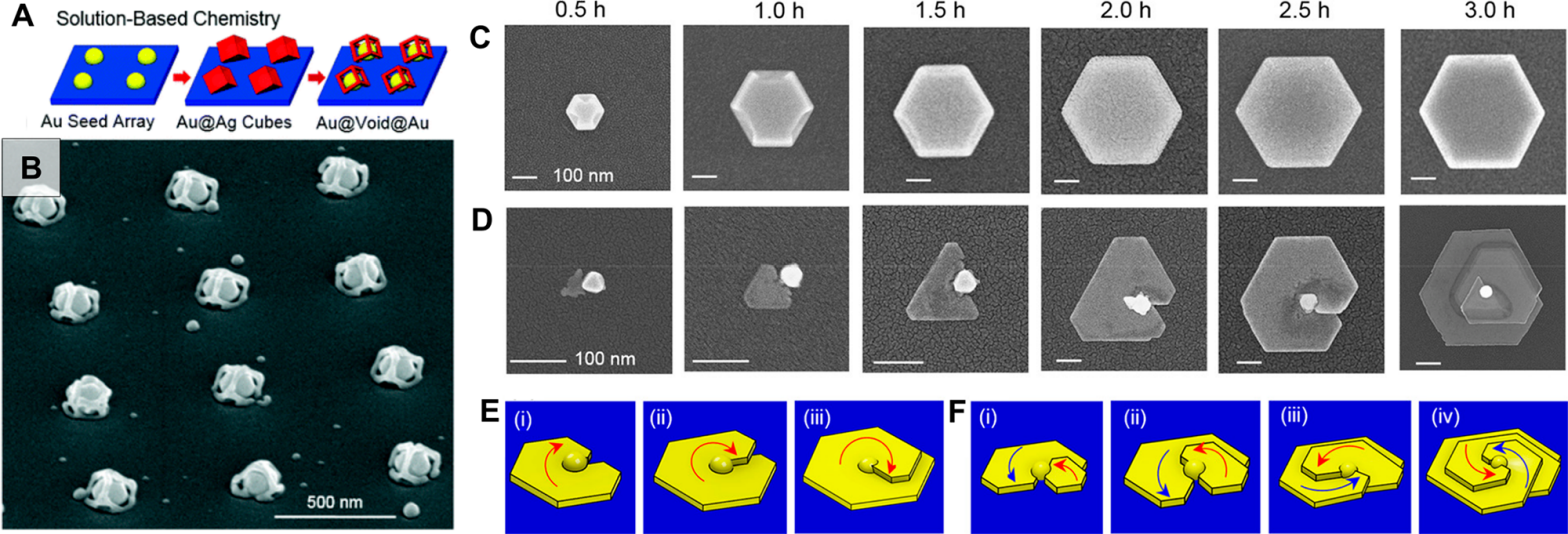
(A) Schematic showing solution-based method for growing Au@void@Au nanoparticles and (B) scanning electron microscopy image of the cage arrays [adapted with permission from ref (194). Copyright 2018 Royal Society of Chemistry.]. (C) Time evolution of growth of a standard hexagonal nanoplate and (D) growth of the plate when protonated phenyl-modified carbon nitride is added as a shape directing agent. Schematics depicting the growth of (E) single and (F) double spirals [adapted with permission from ref (333). Copyright 2021 American Chemical Society].

*In situ* growth is potentially amenable to the fabrication of substrate-truncated structures, as well. Although not purely by *in situ* growth techniques, truncated gold, silver, copper, and palladium nanoparticle arrays have been prepared by dynamic templating and dewetting, where the deposited thin metal film is restructured into a thermodynamically favored shape at high temperatures.^196,340^ Overgrowth of dewetted seed arrays produced faceted truncated structures resembling a Wulff construction (Figure 21D,E). *In situ* growth and colloidal seed deposition have been combined to synthesize shapes with sharp features (other than nanostars) such as concave rhombic dodecahedra (CRDs) and concave cubes (CCs) (Figure 21F–I).^199^ Mirkin’s group found that *in situ* overgrowth gave denser coatings of the nanoparticles on paper substrates compared to attachment of presynthesized CRDs and CCs, which was found to be useful for improved SERS sensing (see Sections 1.3 and 3.3). The synthesis of complex shells, cages (Figure 22A,B), and substrate-oriented chiral structures (Figure 22C–E) has also been established by combining *in situ* overgrowth and top-down dynamic templating.^194,333,334^ In particular, the substrate-oriented chiral structures prepared by plasmon-assisted growth are of special interest for the fabrication of novel biosensors and metamaterials,^38,49^ expressly considering that ordered chiral plasmonic assemblies are not trivial to fabricate starting from colloidal products.^78,86^

## 3 Applications

Since the field of *in situ* grown nanostructures is relatively new, most studies focus on the precise synthesis of such nanomaterials and less on their applications. Thus far, most examples of *in situ* growth result in the fabrication of substrates functionalized with a monolayer of nanoparticles. While BCML-based methods, utilizing multiple steps or by carrying out subsequent top-down lithographic modifications, have produced structures beyond single layers and there are still many potential routes left to be explored for the generation of 3D structures.^39,295^ Thus, in this section, we discuss directions for future applications of *in situ* grown nanomaterials and their contributions toward improving existing nanomaterials for specific applications, with an emphasis on those that can benefit from/utilize plasmonic monolayers.

### 3.1 Plasmonic Metasurfaces

In its most general definition, a metamaterial is an (ordered or disordered) ensemble of subwavelength-sized building blocks whose optical properties are determined by the spatial patterns of each composing unit (or meta-atom) and not by their individual properties. In this regard, plasmonic metamaterials preserve the benefits of plasmonic nanostructures (such as highly enhanced light-matter interactions in the visible electromagnetic range, high surface-to-volume ratio, dynamic tuning of optical properties) but at larger spatial scales.^353^ The rational design of increasingly complex plasmonic architectures enables the combination of SPPs, LSPRs, and SLRs to obtain advanced optical, plasmonic, and photonic behavior. As such, metamaterial engineering creates new perspectives and opportunities owing to their unique structure-dependent properties, implementing the plasmonic properties of nanostructures toward practical applications. Specifically, the exceptional structure-dependent optical properties make plasmonic (and photonic) metamaterials attractive for different fields, such as photocatalysis, biosensing, energy harvesting, and photonics. For example, one recent plasmonic metasurface design attracting attention are nanoparticle superlattices consisting of microscale patch arrays in which every unit is made up of finite nanosized particles.^102−105^ Such structures can be fabricated using soft-lithographic techniques in combination with colloidal synthesis,^354,355^ increasing the number and complexity of the plasmonic response for highly tunable collective optical properties,^356,357^ with promising results for both standard and multiwavelength SERS measurements.^13,86,358−360^ While *in situ* growth generally produces a monolayer of nanoparticles, the implementation of multiple growth steps combined with layer-by-layer fabrication or top-down methods could be utilized to produce 3D plasmonic materials. These combinations could enable the preparation of Moiré lattices, composed by the superposition of two or more layers of plasmonic arrays with controlled twisting angles with respect to one another,^361^ which could be chiral and/or provide highly confined resonances with low optical losses.^362,363^ Such structures have already been realized utilizing BCML-based fabrication.^295^ Next, we discuss some promising potential applications of plasmonic metamaterials.

### 3.2 Plasmon-Based Sensing Applications

To obtain reproducible and quantitative sensing either *via* SERS and/or utilizing SLRs, it is important to fabricate uniform substrates such that quantitative measurements can be performed. As noted above, much work has been done regarding the implementation of *in situ* growth toward the fabrication of sensitive and quantitative SERS sensors.^224,331,339,364^ Recently, it was also demonstrated that uniform and scalable SLRs could be obtained purely utilizing *in situ* growth.^288^ The narrow bandwidth typically associated with SLRs explains the great interest in their implementation into various sensing strategies. The scientific community has shown that SLRs can achieve high spectral sensitivity related to the periodicity of the structures and remarkably high phase sensitivity, features that make these modes ideal candidates for label-free characterization of biomolecular interactions between a target analyte (antigens, DNA, *etc*.) and the corresponding receptor (*e.g.*, antibody, DNA capture, protein, *etc*.).^365−369^

Kravets and co-workers exploited SLRs of gold nanoresonator arrays for the development of chemical sensors and biosensors based on refractive index monitoring, with a phase sensitivity 1 order of magnitude higher than that of the SPP sensors based on the Kretschmann configuration.^370^ In a follow-up study, they proposed Au double-dots arrays on glass covered by a weakly hydrogenated graphene crystal as plasmonic resonators, showing high intensity variation of the reflected light accompanied by extremely fast phase changes, suggesting that plasmonic devices can be employed for molecular recognition and provide high sensitivity at the single-molecule level.^371^ Hanham et al. proposed an alternative sensor-on-chip approach to liquid sensing in the THz regime by using an array of metallic rectangular antennas fabricated on a polyethylene naphthalate substrate.^372^

### 3.3 Surface-Enhanced Spectroscopies

Spectroscopic measurements of the vibrational spectra of molecules or solids enable their unambiguous chemical identification due to the high specificity of vibrational energies. The most common vibrational optical characterization techniques are IR spectroscopy and Raman spectroscopy. These processes involve the energy transfer between an incident photon and the lattice excitations of a solid or vibrational levels of a molecule. Molecular vibrations are IR active if their excitation causes a change in their dipole moment, while they are Raman active if they are associated with a change in the polarizability tensor.^373^ Unfortunately, both IR and Raman spectroscopy suffer from low sensitivities. The IR vibrational cross sections are quite small due to the size mismatch between typical molecules (on the order of nanometers) and IR wavelengths (on the order of microns), while Raman intensities are inherently weak, because they originate from nonlinear scattering phenomena. To achieve the highest possible sensitivity, optical antennas have been used to enhance local electromagnetic fields in their hotspots (tight gaps between adjacent antennas) and to increase the energy transfer between photons and molecular vibrations or phonons in crystals.^374^

**Figure 23:**
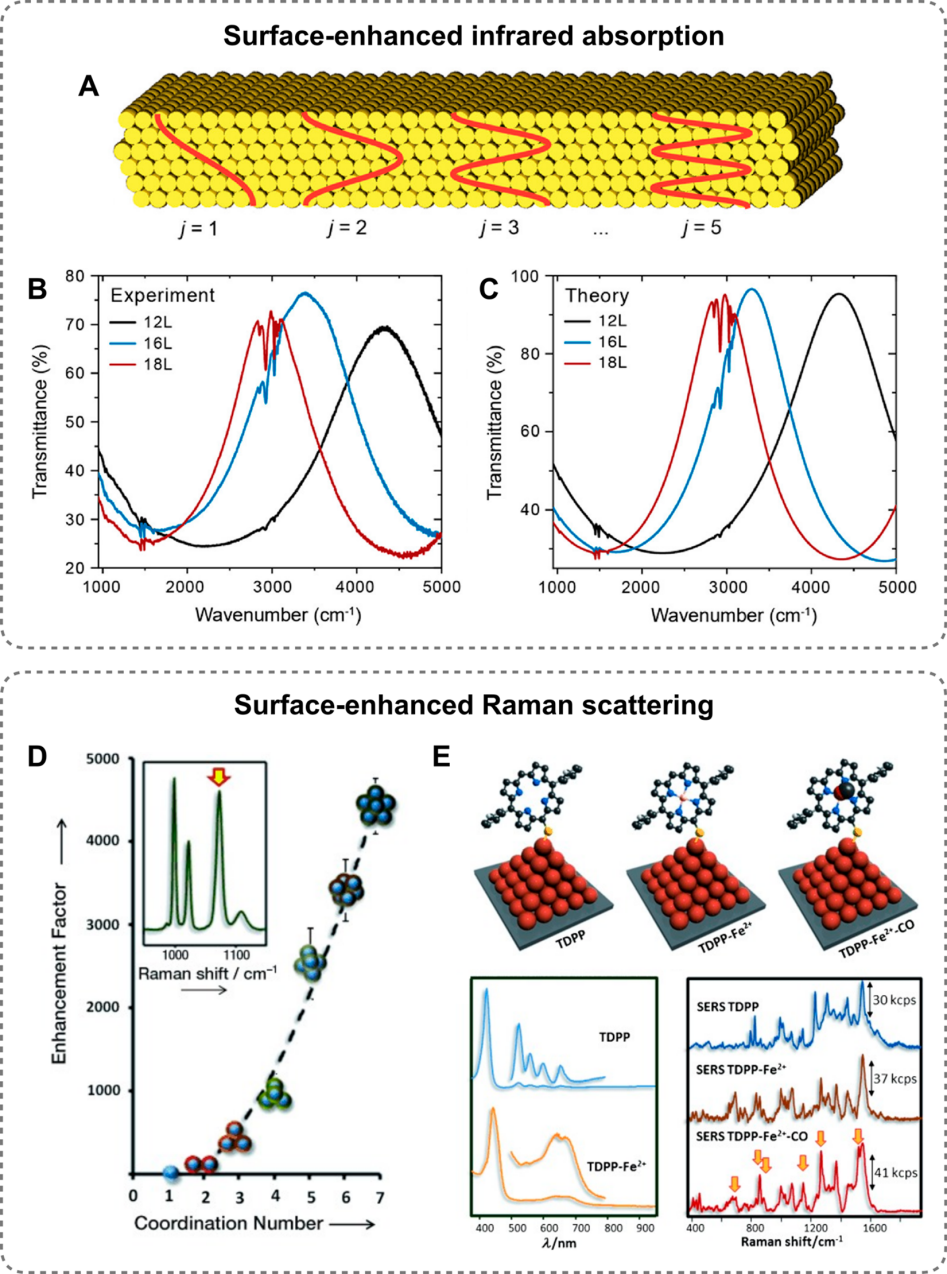
Surface-enhanced infrared absorption (SEIRA) and surface-enhanced Raman scattering (SERS) with 2D and 3D metamaterials. (A) The standing waves supported inside the Au plasmonic supercrystals, which determines the absorption of the metamaterial. *j* denotes the number of nodes of the standing wave. (B) Experimental and (C) theoretical transmittance spectrum of the 3D metamaterial with 12, 16, and 18 number of Au nanoparticle layers which determines the absorption enhancement factor of polystyrene ligand molecules [adapted from ref (377) with permission. Copyright 2021 American Chemical Society under CC BY-NC-ND 4.0]. (D) Increase of the SERS enhancement factor with the number of meta-atoms in the 3D metamaterial. The enhancement factor was determined from the SERS spectrum of benzenethiol (inset) [adapted with permission from ref (385). Copyright 2012 Wiley VCH under CC BY-NC-ND 4.0]. (E) Monitoring the adsorption of CO on the iron porphyrin (TDPP) on macroscale metamaterials. The SERS clearly identify the free adsorbed porphyrin (TDPP), the Fe^2+^-complexed porphyrin (TDPP+Fe^2+^) and the CO adsorption of TDPP-Fe^2+^ [adapted with permission from ref (380). Copyright 2013 Wiley VCH under CC BY-NC-ND 4.0].

In SEIRA, the direct IR absorption of target molecules is enhanced by concentrating the local near field around the surface of optical antennas resonating in the mid-IR. Metasurfaces have recently been extensively explored for SEIRA to generate enhancement over large areas. The ratio between the field amplitude with and without the antenna can reach values of 10–100 in the hotspots, depending on the detailed geometry. The vibrational signal is enhanced by a factor of |*E*/*E*_0_|^2^,^375^ providing SEIRA enhancements exceeding 10^4^ in best-case scenarios.^376^ Most metals behave similarly in the mid-IR, much below the plasma frequency and interband transitions, where they are characterized by large negative values of the real part of the dielectric function and low penetration depths. Mueller et al. created plasmonic metamaterials for SEIRA, Au supercrystals with different Au nanoparticle layers through self-assembly on a liquid subphase (Figure 23A–C).^377^ By varying the numbers of Au layers in the metamaterial, they tuned the absorption, attaining SEIRA enhancement factors of ∼5 × 10^2^ for polystyrene molecules. Nonetheless, despite the great advantages of SEIRA, one major problem in practical applications is the stability of the enhancing substrate. For example, gold nanostructures have low adherence to glass or silicon substrates; they detach readily during the measurements. This problem is especially acute in electrochemical experiments coupled to SEIRA, in which the plasmonic substrate must be stable over a wide range of potentials and electrolytes. In electron beam lithography (EBL) samples, the solution toward this problem is the addition of an adhesion layer (usually ∼3 nm of Cr or Ti). However, the adhesion layer can often prove to be detrimental toward the sensitivity of SEIRA since it adds to the overall thickness of the substrate and the IR light cannot penetrate the entire substrate and reach the sample. On the contrary, for *in situ* grown nanostructures, the adherence of the plasmonic structures to the substrate can be tuned chemically by choosing the optimum molecular ink that favors the *in situ* growth of the nanostructures.

While in SEIRA the signal enhancement due to the presence of an optical antenna scales as |*E*/*E*_0_|^2^, in SERS the scaling goes as |*E*/*E*_0_|^4^, enabling the realization of much higher enhancement factors. The reason for the |*E*/*E*_0_|^4^ dependence can be intuitively understood as follows:^373^ if we consider only the local field enhancement (excitation), |*E*/*E*_0_|^2^, and radiative enhancement (re-emission), we have a SERS enhancement factor scaling with *M*_*Loc*_(*ω*_*L*_)*M*_*rad*_(*ω*_*R*_), where *M*_*Loc*_(*ω*_*L*_) is the enhancement of the local electromagnetic field at the excitation frequency, *ω*_*L*_, and *M*_*rad*_(*ω*_*R*_) is the radiative enhancement at the frequency *ω*_*R*_, in the direction of detection. Usually, *M*_*Loc*_ can be solved relatively well, which yields the local electromagnetic field everywhere. *M*_*rad*_ on the other hand, is much more difficult to solve, and is therefore assumed that the radiative enhancement equals the excitation enhancement; taking into account that the Raman shift is usually small (*i.e.*, *ω*_*L*_ ≈ *ω*_*R*_), we obtain the common approximation:
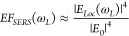
19

Such SERS active plasmonic metasurfaces have been investigated in flexible electronic devices for ultralow detection of biomolecules,^378,379^ as well as for the adsorption of CO on molecular catalysts.^380^ Supercluster metamaterial^381^ nanoparticles and plasmonic supercrystals^377,382^ were explored for SERS due to their high density of hot spots and confined optical modes inside the structures (Figure 23D). In order to obtain optimal enhancements, plasmonic metasurfaces can be designed to enhance both excitation and emission processes in SERS precisely by supporting resonances at both frequencies. Fabrication efforts have been directed toward the realization of controlled large-scale methods to achieve millimeter- and micron-scale plasmonic substrates with nanometric gaps for high SERS enhancement and low fabrication costs. A special feature and benefit that has been investigated with metasurfaces for SERS is the broadband design, which makes the platforms more reproducible over a wide spectral range and widely applicable to many different target molecules, a key aspect in SERS. In this regard, *in situ* growth methods provide an excellent platform for the reproducible fabrication of large-scale SERS substrates, with high enhancement factors and high densities of hot spots.

Besides the electromagnetic enhancement factors, another crucial aspect of SERS substrates (often ignored) is the surface dynamics of the target analyte and the capping agent.^383^ As a rule of thumb, if the target analyte has a lower adsorption energy than that of the capping agent, it cannot adsorb close to the metal surface, and thus the SERS signal will be weak, despite the high electromagnetic enhancement provided by the nanostructures. As highlighted above, *in situ* synthesis methods can avoid this problem since they do not require the use of stabilizing/capping agents. In principle, EBL could also produce nanostructures without any capping or stabilizing agents, however, *in situ* grown structures have two clear advantages in this regard: higher throughput and, importantly, higher Q factors. Chemically synthesized nanostructures have higher Q factors than their lithographically synthesized counterparts due to better crystallinity.^384^ Therefore, for both SERS and SEIRA applications, *in situ* grown nanostructures combine the advantages of top-down synthesis methods regarding the surface chemistry, and the advantages of chemical synthesis, in terms of the higher crystallinity (and higher *Q* factor) of the nanostructures and the dynamic tuning of the nanostructure morphology.

### 3.4 Chiral Plasmonic Metasurfaces

Chiral plasmonic metasurfaces, where the array units are either composed of chiral elements or arranged into chiral superstructures, are receiving increasing attention, as new synthetic possibilities emerge for the preparation of enantiomerically pure plasmonic nanoparticles.^40,149,150^ The optical responses of these systems depend on the right or left circular polarization of the incident light, mimicking characteristics observed in nature, for example, for antibodies, viruses, exosomes, and other nanostructures.^40^ Circular dichroism (CD) measures the extinction difference between the right and left circularly polarized light. The optical activity for these systems is measured through the anisotropy factor, *g* (Δε/ε), where Δε represents the molar circular dichroism and ε the molar extinction.^386^ Yu et al. generated high-quality circularly polarized light over a wide range of wavelengths, suitable for random orientation of the incident linear polarization, by using quarter-wave plate metasurfaces. The light generated had high degrees of circular polarization (>0.97) over a range of wavelengths from 5 to 12 μm.^387^ More recently, Goerlitzer and co-workers have studied the optical chiral response of 3D-metasurfaces made by core–shell particles self-assembled into crescent nanostructures with large lattice periods. The crescent shape of the array components does not affect the diffractive states arising from the system, but leads to excitation of intrinsically chiral SLRs that selectively respond to incident circularly polarized light.^38^ Chiral plasmonic metamaterials are especially useful for biological sensing due to the chiral nature of biologically and medically relevant systems, such as amino acids, proteins, and DNA, and therapeutics.^49,56,388,389^

### 3.5 Plasmonic Lasers

Plasmonic-based lasing emission holds potential for manufacturing nanoscale light sources and biosensing. Lasing action *via* SLRs exploit the resonances as optical cavities, which compensate optical losses and enhance the localized electromagnetic field, inducing strong enhancements in the gain media emission.^390^ The light emission of fluorophores in proximity to the periodic plasmonic arrays composed of plasmonic antennas can be significantly modified. Hybridization of the localized plasmonic modes with photonic states generates increased density of the optical states, providing preferential radiative decay for the excited molecules, which results in enhanced and highly directional emissions.^391^ Odom’s group demonstrated lasing action from band-edge modes in arrays of silver nanoparticles surrounded by a homogeneous media.^392^ They also demonstrated real-time modification of plasmonic laser spectral output by modifying the refractive index of the media in a microfluidic setup.^393^ Apart from the emission wavelength, another advantage of a SLR-based lasing architecture is the possibility of controlling emission direction and polarization, modifying the lattice period, accessing lattice high symmetry points (Γ or M) that overlap with quantum dot emission.^394^ By exploiting hybrid waveguided SLR modes arising from the difference in refractive index among the emitter layer and the substrate, it is possible to engineer the polarization of the lasing emission (radially or azimuthally).^395^

### 3.6 Photocatalysis

While for these applications top-down nanofabrication of the nanostructures is common, the relatively simple and high-throughput synthesis of *in situ* grown nanostructures opens a wealth of possible applications in both photocatalysis and surface-enhanced sensing. Thus far, significant developments have been made toward applying plasmonic materials toward directing catalyst driven reactions, such as H_2_ and O_2_ dissociation^75,396^ or reactions relevant to environmental remediation.^397,398^ Plasmonic nanocrystals^27,28,399,400^ and substrates/electrodes functionalized with plasmonic nanostructures or nanoantennas prepared by top-down nanofabrication, such as those explored by the groups of Halas and Nordlander,^396,401,402^ Cortés,^403,404^ Atwater,^405^ and others^406,407^ are some popular examples of platforms for probing and optimizing plasmon-enhanced catalysis. Metamaterials have recently emerged as a step forward toward translating the potential of plasmonic nanostructures into practical, large-scale, photocatalytic applications.^3,76^ Narang and co-workers have explored plasmonically enhanced chemistry in a number of configurations and reactions.^75,405,408,409^ Two-dimensional metamaterials (*i.e.*, metasurfaces) provide extensive platforms to engineer photocatalytic materials, enhancing their solar light absorption capacity and the consequent hot-carrier generation. They have high surface areas, offering plentiful catalytic active sites for surface reactions. In most cases, metasurfaces are synthesized using top-down fabrication methods, such as EBL. However, such methods are time-consuming, requiring hours or even days, for the fabrication of large-scale metasurfaces and expensive cleanroom facilities.^410^ On the other hand, *in situ* growth methods offer high-throughput solutions to synthesizing metasurfaces. In principle, nonpatterned *in situ* grown plasmonic surfaces can be rapidly synthesized on meter scales^411^ (Section 2.2), while for patterned *in situ* grown metasurfaces the scalability depends on the particular method (Section 2.3).

**Figure 24:**
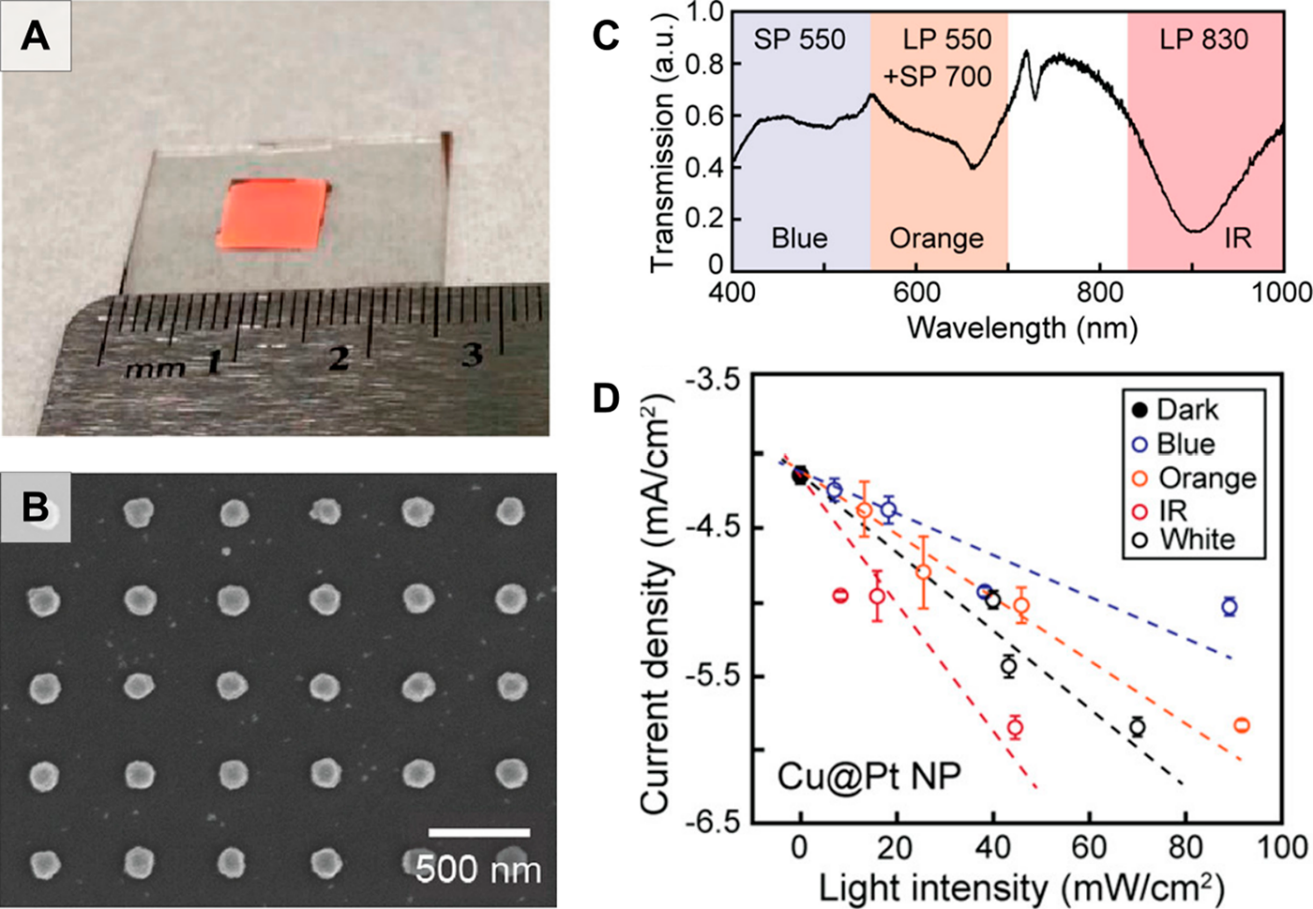
Plasmonic metasurfaces for photocatalysis. (A) Optical image and (B) scanning electron microscopy (SEM) of the synthesized Cu–Pt bimetallic metasurface. (C) Experimental transmission spectrum of the Cu–Pt metasurface, highlighting the different lattice plasmon (LP) and surface plasmon (SP) resonances from the visible to the infrared (IR). (D) Photocatalytic HER voltammogram of the Cu–Pt metasurface under different irradiation wavelengths. Current density *vs* light intensity of Cu@Pt NP metasurface at a potential (*vs* RHE) of −0.2 V [adapted with permission from ref (413). Copyright 2021 American Chemical Society].

Another approach in photonic engineering of metasurfaces for photocatalysis comes from the utilization of SLRs in both metal and dielectric materials (Figure 24A–C, Section 1.4). Several groups have demonstrated higher activities in hydrogen evolution reactions (HER) when catalytic nanostructures are formed into metasurfaces that can sustain SLRs.^412^ Through a 2D array of bimetallic Cu–Pt core–shell nanoparticle lattices, Deng et al. showed that the largest photocurrent densities take place under near-IR illumination.^413^ The SLRs were shown to be the main contributor to the observed photocurrent enhancement under white light illumination, surpassing LSPR excitations with 2-fold improvement in HER catalytic activity because of greater light absorption and highly confined electromagnetic fields exhibited by the lattice modes (Figure 24D). These bimetallic systems are expected to act as good photocatalysts for HER due to the combination of highly catalytically active Pt sites with strongly plasmonic effects of Cu, with similar examples being found for other bimetallic systems.^414^ In addition to the interband transitions and the LSPR modes of single Cu particles, the lattices are engineered to exhibit a strong SLR mode in the near-IR region to extend the absorbed solar energy range of the material. Excitation of either mode is expected to generate hot carriers on plasmonic Cu, which can then be injected on the overlaying Pt shell.

In a similar study, Gao et al. fabricated a hybrid metal–semiconductor structure in which an ultrathin layer of Fe_2_O_3_ is deposited on a Au nanopillar superlattice.^415^ Photocurrent was enhanced up to 50% for the oxygen evolution reaction (OER) due to the broadband absorption of the hybrid structure, arising from the combination of LSPR and collective photonic modes of the superlattice. In a parallel study, Fe_2_O_3_ nanorods were grown on Au nanohole arrays.^416^ This architecture can produce even larger photocurrent increases (∼10 fold) for OER, as the nanowires act similarly to an optical fiber when the collective modes are excited, further enhancing the absorption in Fe_2_O_3_. Both examples demonstrate how limitations due to the short charge carrier diffusion length of Fe_2_O_3_ can be side-stepped by using carefully engineered plasmonic nanostructures and generating these charges close to the surface of the catalytic material. In this regard, new synthesis methods, such as *in situ* growth, could prove valuable for moving from photocatalytic proof-of-concept demonstrations to practical applications. *In situ* grown hybrid metasurfaces offer robust platforms for incorporating plasmonic nanostructures, prepared with basic laboratory equipment and at high throughput, with improved absorption in the visible part of the electromagnetic spectrum, catalytically active materials (*e.g.*, Pt or Pd) and with different optical resonances, such as SPR and SLR.^288^

One additional advantage of *in situ* grown nanostructures is greater control over surface chemistry and the dynamics of the chemical reactants and intermediates. If the surfaces of the nanostructures are covered with strongly adsorbed ligands from the synthesis or self-assembly process (such as thiols, oleylamine), then there will be fewer available surface catalytic sites, thus affecting the photocatalytic efficiency.^285^ This difference makes *in situ* growth methods stand out compared to self-assembly methods, for example, in which strongly adsorbed capping agents are used to improve the stability of colloidal nanostructures during the self-assembly process.^381^ While the nanostructures can be “stabilized” following growth to maintain fixed positions on the substrate (as described in Section 2.5), in principle, the *in situ* grown structures do not require stabilizing surfactants; therefore, it should be possible to make the entire surface of *in situ* grown nanostructures available for catalyzing photoreactions. This aspect is also relevant to sensing applications (see Section 3.3).

### 3.7 Photovoltaics

Plasmonic metasurfaces find applications as thin-film solar cells, since surface roughness represents a major source of carrier recombination in the surface and junction regions. By using nanostructured surfaces, it is possible to overcome these limitations by reducing the physical thickness of the cell while maintaining the desired scattering effect used to increase the effective path length of the incoming light.^417^ In fact, metallic nanoparticles can be used as scattering elements to trap propagating plane waves from the sun and to direct the light into a thin absorber layer. In this scenario, nanoparticles act as nanoantennas by coupling the plasmonic near field to a surrounding semiconductor material, which increases its absorption cross-section.^417−419^ Moreover, sunlight can couple to the SPP modes supported at the metal/semiconductor interface, increasing the amount of photocarriers in the semiconductor.^417,420^ Catchpole and Polman demonstrated that the shape and size of the scatterers (Ag or Au particle) represent key parameters for designing plasmonically enhanced solar cells, leading to effective path length enhancement due to variation of the near-field coupling.^421^ Ferry et al. used a hydrogenated amorphous Si (a-Si:H) solar cell by exploiting plasmonic light trapping structures built into the metallic back contact: the light is scattered into guided modes within the thin-film cell, providing considerable enhancements of the photocurrent for a given cell thickness.^419^ The concept of guiding and highly concentrating light *via* plasmonic nanostructures in these types of architectures open up new opportunities for the design of solar cells where light is fully absorbed in a gain medium composed of a single layer or even a single molecule.^417,422^

### 3.8 Metasurfaces for Photon–Photon Conversion

Ultrathin metasurfaces with subwavelength thickness open alternative means of achieving efficient nonlinear optical interactions due to the presence of strong field enhancements in subwavelength meta-atoms, which enables shaping the nonlinear tensor and boosting the efficiency of different nonlinear processes. New materials, which exhibit strong bulk nonlinearities, such as multiquantum-wells (MQWs), must meet specific polarization conditions.^423^ The transitions are polarized in the direction perpendicular to the layers, and therefore they are not accessible by plane waves at normal incidence. Plasmonic nanoantennas and metasurfaces on top of MQWs have been proposed to couple excitation light normal to the structure into longitudinal electric fields within the MQWs.^424^ As an example, asymmetric gold cross nanoantennas on top of a MQW heterostructure enable efficient light coupling to intersubband transitions in MQWs with second harmonic generation (SHG) efficiency of almost 2 × 10^–6^ using a pump intensity of only 15 kW cm^–2^.^423^

An alternative to stacking multiple layers in MQWs, ultrathin metasurfaces provide another promising and rapidly growing field targeting efficient nonlinear generation. Unlike 3D bulk structures, nonlinear optical metasurfaces (NOMs) can exhibit strong nonlinear optical responses at ultrathin thicknesses (much smaller than the operating wavelengths), removing phase-matching requirements. Surface plasmons supported at metal–dielectric interfaces offer an efficient approach for enhancing nonlinear light–matter interactions due to strong light confinement.^425^ The advantage of *in situ* grown metasurfaces is that the shape of plasmonic structures (*i.e.*, meta-atoms) can be tailored easily and rapidly (see Section 2.6), through chemical growth, to enhance light–matter interactions resonantly at different frequencies, boosting the efficiency of nonlinear generation.

### 3.9 Strong Coupling

Light–matter interactions promoted by the resonant behavior of plasmonic nanoparticles can establish strong^426−429^ and ultrastrong^430,431^ coupling at room temperature. These interactions are proportional to the square root of the number of quantum emitters participating in the interaction, and their associated dipole moment.^432^ The most characteristic optical feature of strongly coupled systems is the appearance of a Rabi splitting as a direct consequence of a coherent energy transfer leading to the formation of two polaritonic states.^432^ Väkeväinen et al. demonstrated the possibility of reaching this regime using SLRs as tunable optical cavities for a common organic dye molecule.^433^ Both the measured and simulated spectra display the characteristic bending and anticrossing features as a function of the emitter concentration. Here, SLRs were exploited for their collective nature, combining long spatial and time coherence with high local electric fields (corresponding to small mode volumes) to promote strong-coupling interactions with the emitter molecules in proximity to the nanoparticle surface.

### 3.10 Photothermal Applications

The field of thermoplasmonics (*i.e.*, converting light energy to thermal energy through plasmonic structures) addresses applications such as photothermal therapy and drug delivery, thermal optical data storage or solar thermal energy among others.^434^ Nanoplasmonic metamaterial absorbers offer a route for low-cost and compact designs of practical thermal platforms for solar energy harvesting, while maintaining a high optical cross-section due to the plasmon resonances sustained in the visible range of the electromagnetic spectrum. At the same time, coupling between the individual nanostructures in a metamaterial gives rise to collective resonances, creating almost perfect absorbers. As a rule of thumb, for attaining a high absorbance, and therefore high light-to-heat conversion yields, the radiative decay of the photoexcited plasmon and lattice resonances of the metamaterials must be suppressed. High light-to-heat conversion can be facilitated by either decreasing the size of the individual metal meta-atoms (the radiative scattering scales with the volume of the nanostructure) or by changing the dielectric function of the material.

**Figure 25:**
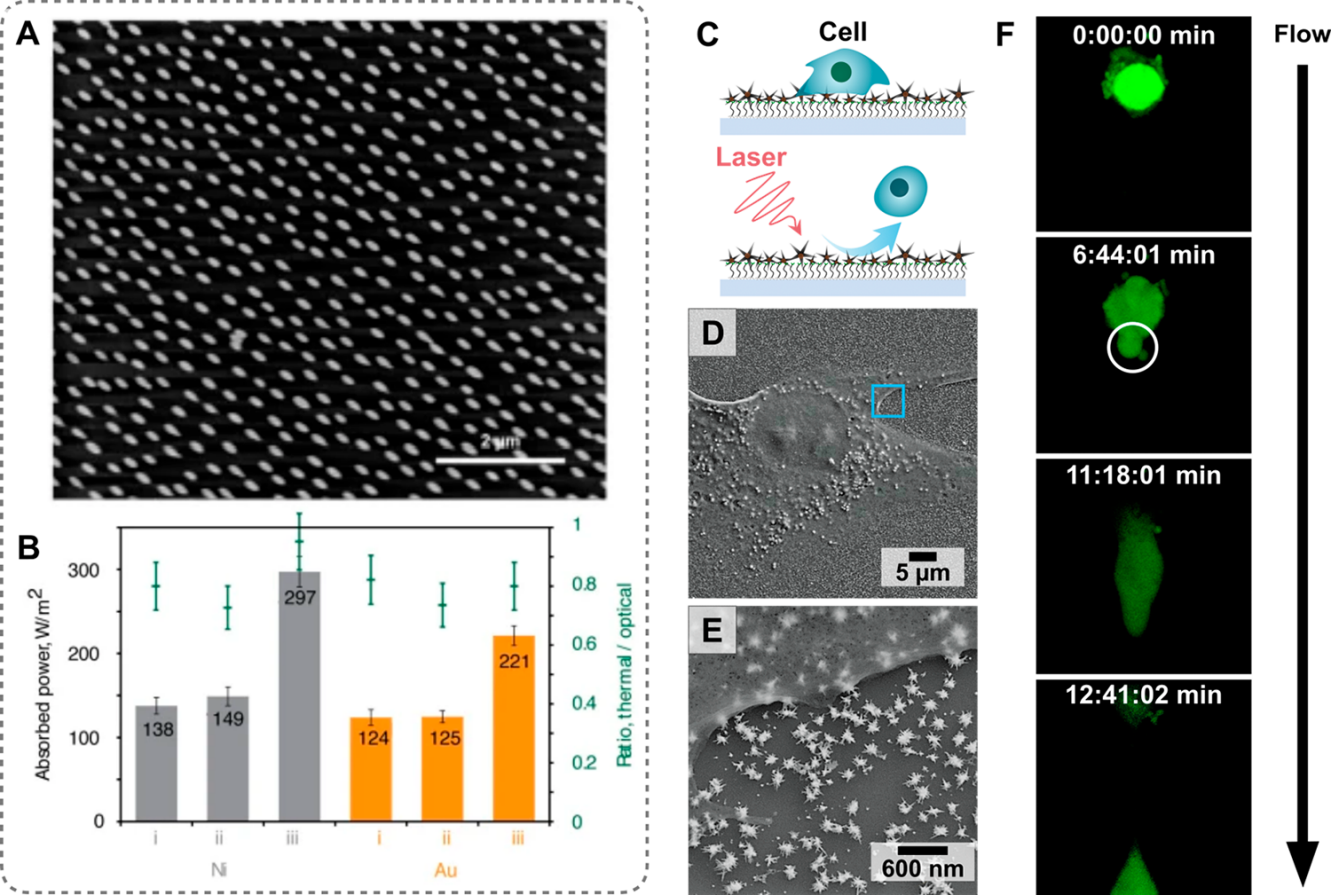
Light-to-heat conversion by plasmonic metasurfaces. (A) Scanning electron microscopy (SEM) image of a typical Au metasurface (or Ni, which appears similarly), prepared by hole mask colloidal lithography. (B) The light-to-heat conversion yield of Au and Ni metasurfaces with different-sized meta-atoms: (i) 60 nm × 84 nm, (ii) 80 nm × 112 nm, and (iii) 100 nm × 140 nm [adapted with permission from ref (435) Copyright 2014 Nature Publishing Group under CC BY-NC-ND 3.0]. (C) Schematic showing a glioblastoma cell interacting with a thermoplasmonic substrate. (D,E) SEM images of a cell on a thermoplasmonic nanostar-coated substrate. (F) Laser scanning confocal microscopy image of the removal of a grafted cell using plasmonic heating from the *in situ* overgrown structures [adapted with permission from ref (6). Copyright 2020 American Chemical Society under CC BY-NC-ND 4.0].

While initial studies in thermoplasmonics have focused on the intricate design of individual nanoplasmonic units, through time-intensive theoretical and fabrication methods, the focus has shifted toward scalability and affordability. Increasingly, new synthetic methods that can yield large-scale and high-absorbance metasurfaces for photothermal applications have been explored. For example, Jonsson et al. have designed plasmonic metasurfaces of Ni and Au, through hole mask colloidal lithography, on centimeter-scale areas, with approximately 80% light-to-thermal energy conversion (Figure 25).^435^ These plasmonic metasurfaces achieved similar, if not larger, light-to-thermal conversion yields to standard absorber materials, *e.g.*, a film of amorphous carbon.

Another exciting application of plasmonic metasurfaces, based on light-to-heat conversion, is the development of antifogging transparent metasurfaces. Surface fogging negatively affects many materials, such as windshields, cameras, or electronic displays. Traditional approaches for developing antifogging transparent surfaces focus on designing superhydrophilic or superhydrophobic coatings. However, Walker et al. showed that bimetallic Au-TiO_2_ transparent plasmonic metasurfaces yielded significantly better results than state-of-the-art antifogging coatings.^436^ The Au-TiO_2_ metasurface works by capturing solar light and converting it to heat, delaying the fogging and decreasing the fogging time by up to 4× compared to the state-of-the-art antifogging coatings. Moreover, such plasmonic metasurfaces also decrease the nucleation of condensates.

Metasurfaces have become increasingly popular in light-to-heat conversion applications. *In situ* grown plasmonic metasurfaces have the potential to expand the possibilities of thermoplasmonics by increasing the scalability of such plasmonic platforms and improving the dynamic tunability of the individual meta-atoms, which influences the optical absorption of the metasurface. Thus, for example, the same *in situ* grown metasurface could be “recycled” or reused for different applications, by dynamically changing its optical absorption from the IR range to the visible or *vice versa* with high throughput. Furthermore, since the nanoparticles are fixed on the substrate and colloidal stability is not a factor, “cleaning” and removal or replacement of the capping ligands is more feasible, making these substrates more amenable for biological applications. Consequently, anisotropic thermoplasmonic structures such as those presented in Sections 2.6.1 and 2.6.3 prepared by *in situ* growth or *in situ* seed overgrowth have thus far primarily been applied for biological applications, such as devices for cell isolation/capture and recollection (Figure 25).^6,332^

### 3.11 Optical Tweezers

The positions of nanoobjects can be controlled with nanoscale precision using optical tweezers, a powerful tool that enables object trapping in the proximity of the focus of a laser beam.^437^ Conventionally, the trapped volume is diffraction limited and significant Brownian motion of the trapped nanoparticles translates into the need for high laser power to achieve stable trapping.^438^ Grigorenko et al. reported the experimental realization of a 3D nanometric optical tweezer using gold nanodots to fabricate an array of optical traps. They exploit the near field to shrink the trapping volume beyond the diffraction limit and to quench Brownian motion of the trapped nanoparticles by nearly an order of magnitude compared to conventional tweezers operating under same conditions.^439^

## 4 Summary and Prospects

As described above, there are a plethora of opportunities for the *in situ* growth and overgrowth of gold nanoparticles on substrates like polymers, oxides, 2D materials, and semiconductors. While some of the methods developed have been inspired by colloidal synthesis to modify particle morphology (*i.e*., controlling the balance of reagents to induce symmetry breaking, favor the formation of thermodynamic *vs* kinetically favored products, *etc.*), there are significant differences between *in situ* and colloidal environments that affect anisotropic growth. Additional synthetic studies will help elevate this class of approaches to the same relevance as colloidal techniques for manufacturing plasmonic materials and devices. Based on the current progress of the field, important synthetic parameters that should be considered for *in situ* growth schemes include:

1. Competition between spontaneous colloidal nucleation and substrate growth (secondary nucleation).
2. Surface density of seeds or nucleation sites.
3. Interaction strength between the nanoparticles/seeds and the substrate.
4. The chemical environment (constituents of the growth solution, pH and temperature, *etc*.).
5. Seed size, crystal structure, orientation on the substrate.

Of these factors, precisely controlling the location and density of nucleation sites (analogous to controlling the seed concentration in colloidal synthesis), as is done with BCML and other patterning techniques, is especially important for controlling the concentration of nuclei for performing synthetic investigations. Moreover, preventing the formation of nuclei in the growth solution away from the substrate or from intended nucleation/growth sites is vital. Note that mass transport for substrate growth differs from 3D systems, so these differences must be taken into consideration when developing models for these systems. The use of different flow profiles for mass transport to the substrate also offers an interesting avenue for controlling *in situ* growth.^6,304,440^ Competition between *in situ* growth and colloidal nucleation unpredictably consumes reagents and creates nuclei that can deposit in unintended locations. Some strategies for preventing or reducing homogeneous or secondary nucleation include:

1. Implementing only weak reductants in the growth solution and using substrate bound or native strong reductants.
2. Using overgrowth solutions with slower growth kinetics.
3. Reducing interactions of the growth solution with other surfaces (*e.g*., glass).
4. Performing overgrowth from smaller (higher surface energy) seeds.
5. Using clean water sources.

With colloidal syntheses, TEM characterization (including tomography scanning, and high-resolution TEM) provides key insight into the growth by providing crystallographic and morphological information.^441^ Instead, *in situ* growth characterization relies mainly on SEM and scanning probe microscopy, since the nanoparticles are fixed on substrates. Consequently, obtaining the detailed crystallographic and structural information necessary for understanding growth (especially when working with nanoparticles having diameters of a few nanometers) is difficult. Some of the critical contributions to *in situ* syntheses were elucidated with high-resolution TEM characterization, which helped to provide important insight. For example, Kumar’s sample preparation directly on custom-made TEM grids coated with a thin film of silicon nitride enabled characterization of gold nanoparticles down to ∼5 nm.^198^ Focused ion beam milling can also be used to prepare the samples for high-resolution TEM.^202,204^ Characterization can also be performed by removing the nanostructures from the substrate, as was done for syntheses of standing nanowires.^204^ Liquid-phase TEM (LPTEM) can also offer insights into substrate-grown structures, especially on carbon thin films or 2D materials. In this setting, the generation of radiolytic species may interfere with the formation of nanomaterials *in situ*, it can also be leveraged to adjust the reaction kinetics.^442,443^ Nevertheless, substrates compatible with LPTEM are restricted to electron-beam transparent thin films, and mass transport of growth reagents to the substrate would differ from larger-scale systems since the growth solution is confined to a thin liquid layer.

Regarding optical characterization, there are also challenges with sufficient spatial resolution to characterize the substrate-bound nanoparticles. For instance, UV–visible spectroscopy and small-angle X-ray scattering are common techniques used for colloidal systems. Instead, dark-field spectroscopy and scanning near-field optical microscopy could be used.

Overall, there are tremendous opportunities for further exploration into growth and shape control *in situ*, which can lead to new methodologies for creating substrates with select chemical and physical properties that may be of broad interest to those designing metamaterials, biomedical platforms, sensors, catalysts, and devices. The formation of particles directly on the substrate opens the door to modification of the particle geometries in all directions relative to the surface, where control in the *x*–*y* directions is normally difficult to achieve with self-assembly. Arranged standing nanowires, oriented anisotropic structures (*e.g.*, triangles, platelets, nanostars, chiral nanohooks^444^), truncated particles,^196^ and oriented dimers,^98^ are some examples of arrangements that can be prepared *in situ*. Some of these structures and 3D constructions could be achieved using multiple growth steps, layer-by-layer fabrication, or by combining *in situ* growth with top-down methods, and some works related to BCML have explored this possibility.^39,295^ In one example, Jeong et al. fabricate oriented dimers with controlled spacings by combining BCML with glancing angle physical vapor deposition.^98^ Furthermore, plasmonic nanoparticle-2D material heterostructures are also if interest, and could be fabricated through subsequent growth steps, as demonstrated by Li et al.^445^

As with colloidal syntheses, the components of the growth solution can be modified to optimize the yield of a desired structure. The next generation of *in situ* synthetic protocols can leverage the factors that set *in situ* growth apart from colloidal synthesis. For instance, a major opportunity of *in situ* growth lies in the fact that the particles are fixed or bound to the substrate. This constraint means that colloidal stability and solvent compatibility do not need to be considered and paves the way for exploring growth in more extreme conditions: high temperature, high concentration of salts, testing extremely low or high pH, surfactant-free synthesis, *etc*. Pursuing the development of surfactant-free synthesis offers a major contribution to this class of techniques, including green and sustainable chemistry, and enabling facile integration with other materials (such as graphene, 2D materials, and other metals) for magnetism, optics, energy, and catalysis applications.^353,446,447^ Furthermore, nanoparticle–substrate interactions offer another handle for modifying morphology and offers versatility accessing new structures. Finally, hybrid structures can be envisioned as an interesting future direction, opening new paths for combining optical, chemical and material properties of different type of nanoparticles growth sequentially *in situ*, for example. While synthetic control of *in situ* growth may not be as developed as colloidal bottom-up synthesis, this method has already produced plasmonic materials that find applications in sensing,^59,197,199,224^ cell culture,^62^ cancer diagnostics,^6^ and electronics/optoelectronics.^39^ The current knowledge base for *in situ* growth and the latest developments in characterizing substrate-bound nanomaterials provides a suitable leaping-off point, which can substantially expand the impact of *in situ* growth.

## Abbreviations

2D: Two-dimensional
3D: Three-dimensional
AA: Ascorbic acid
APTES: (3-Aminopropyl)triethoxysilane
AuNP: Gold nanoparticle
BCML: Block-copolymer micelle lithography
CC: Concave cubes
CRD: Concave rhombic dodecahedra
CTAB/C: Cetyltrimethylammonium bromide/chloride
DFT: Density functional theory
DMAP: 4-Dimethylaminopyridine
DMF: Dimethylformamide
EBL: Electron beam lithography
GGA: Generalized gradient approximation
GO: Graphene oxide
HAADF-STEM: High-angle annular dark-field scanning transmission electron microscopy
HEPES: 4-(2-Hydroxyethyl)-1-piperazineethanesulfonic acid
HER: Hydrogen evolution reaction
IR: Infrared
LSPR: Localized surface plasmon resonance
MBA: 4-Mercaptobenzoic acid
MPTMS: (3-Mercaptopropyl)trimethoxysilane
MQW: Multiquantum wells
NIL: Nanoimprint lithography
NOM: Nonlinear optical material
OER: Oxygen evolution reaction
PAW: Projector augmented wave
PBE: Perdew-Burke-Ernzernhof
PDMS: Polydimethylsiloxane
PNIPAm: Poly(*N*-isopropylacrylamide)
PVP: Polyvinylpyrrolidone
rGO: Reduced graphene oxide
SEIRA: Surface-enhanced infrared absorption
SEM: Scanning electron microscopy
SERS: Surface-enhanced Raman scattering
SHG: Second harmonic generation
SLR: Surface lattice resonance
SPP: Surface plasmon polariton
TEM: Transmission electron microscopy
TMD: Transition metal dichalcogenide
UV: Ultraviolet
VASP: Vienna *ab Initio* Simulation Package
